# Interferon signaling promotes tolerance to chromosomal instability during metastatic evolution in renal cancer

**DOI:** 10.1038/s43018-023-00584-1

**Published:** 2023-06-26

**Authors:** Luigi Perelli, Federica Carbone, Li Zhang, Justin K. Huang, Courtney Le, Hania Khan, Francesca Citron, Edoardo Del Poggetto, Tony Gutschner, Hideo Tomihara, Melinda Soeung, Rosalba Minelli, Sanjana Srinivasan, Michael Peoples, Truong Nguyen Anh Lam, Sebastian Lundgren, Ruohan Xia, Cihui Zhu, Alaa M. T. Mohamed, Jianhua Zhang, Kanishka Sircar, Alessandro Sgambato, JianJun Gao, Eric Jonasch, Giulio F. Draetta, Andrew Futreal, Ziad Bakouny, Eliezer M. Van Allen, Toni Choueiri, Sabina Signoretti, Pavlos Msaouel, Kevin Litchfield, Samra Turajlic, Linghua Wang, Ying Bei Chen, Renzo G. Di Natale, A. Ari Hakimi, Virginia Giuliani, Timothy P. Heffernan, Andrea Viale, Christopher A. Bristow, Nizar M. Tannir, Alessandro Carugo, Giannicola Genovese

**Affiliations:** 1 Department of Genitourinary Medical Oncology, The University of Texas MD Anderson Cancer Center, Houston, TX USA; 2 Nerviano Medical Sciences, NMS Group Spa, Milan, Italy; 3 TRACTION platform, The University of Texas MD Anderson Cancer Center, Houston, TX USA; 4 Department of Genomic Medicine, The University of Texas MD Anderson Cancer Center, Houston, TX USA; 5 Junior Research Group ‘RNA Biology and Pathogenesis’, Medical Faculty, Martin Luther University Halle-Wittenberg, Halle, Germany; 6 Department of Pathology, The University of Texas MD Anderson Cancer Center, Houston, TX USA; 7 Dipartimento Universitario di Medicina e Chirurgia Traslazionale, Università Cattolica del Sacro Cuore, Rome, Italy; 8 Department of Medical Oncology, Dana-Farber Cancer Institute, Boston, MA USA; 9 Department of Oncologic Pathology, Dana-Farber Cancer Institute, Boston, MA USA; 10 Center for Precision Environmental Health, Baylor College of Medicine, Houston, TX USA; 11 The Francis Crick Institute, London, UK; 12 Department of Pathology, Memorial Sloan Kettering Cancer Center, New York, NY USA; 13 Department of Urology, Memorial Sloan Kettering Cancer Center, New York, NY USA; 14 Human Oncology and Pathogenesis Program, Memorial Sloan Kettering Cancer Center, New York, NY USA; 15 Department of Biology, IRBM S.p.A., Rome, Italy; 16 David H. Koch Center for Applied Research of Genitourinary Cancers, The University of Texas MD Anderson Cancer Center, Houston, TX USA

**Keywords:** Renal cell carcinoma, Cancer genomics, Metastasis, Cancer

## Abstract

Molecular routes to metastatic dissemination are critical determinants of aggressive cancers. Through in vivo CRISPR–Cas9 genome editing, we generated somatic mosaic genetically engineered models that faithfully recapitulate metastatic renal tumors. Disruption of 9p21 locus is an evolutionary driver to systemic disease through the rapid acquisition of complex karyotypes in cancer cells. Cross-species analysis revealed that recurrent patterns of copy number variations, including 21q loss and dysregulation of the interferon pathway, are major drivers of metastatic potential. In vitro and in vivo genomic engineering, leveraging loss-of-function studies, along with a model of partial trisomy of chromosome 21q, demonstrated a dosage-dependent effect of the interferon receptor genes cluster as an adaptive mechanism to deleterious chromosomal instability in metastatic progression. This work provides critical knowledge on drivers of renal cell carcinoma progression and defines the primary role of interferon signaling in constraining the propagation of aneuploid clones in cancer evolution.

## Abstract (web summary)

Using genetically engineered models, Genovese and colleagues study patterns of convergent evolution in renal cancer, and pinpoint dysregulation of interferon signaling as a means of adaptation to chromosomal instability in metastatic progression.

## Main

**Extended Data Fig. 1:**
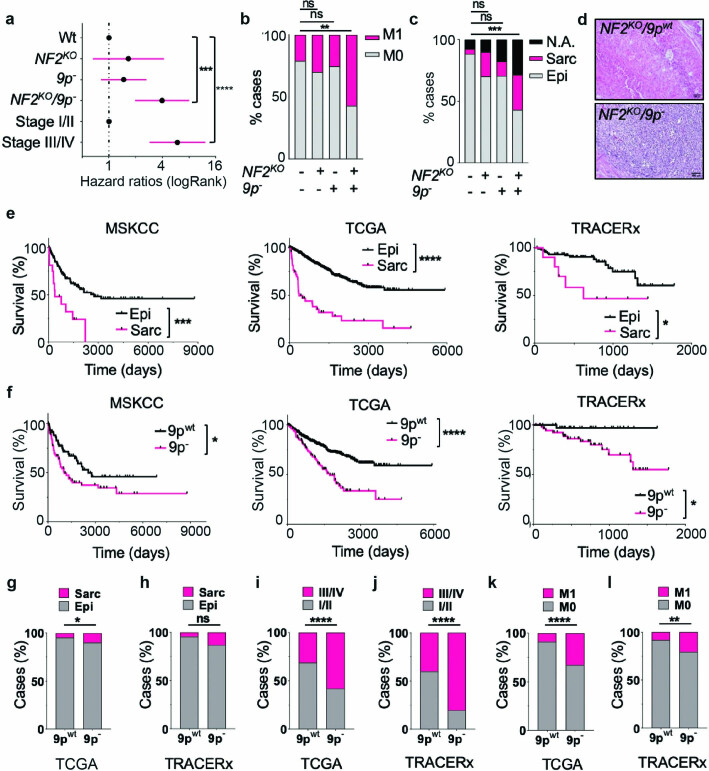
Clinical features of RCC characterized by loss of *9p*. a) Odds plot showing enrichment fatal events in NF2KO/9p^−^ cases and stage III/IV among MSKCC cohort patients, data are represented as hazard ratios with upper and lower limits (N = 134 patients), p values = 2.70*10-4 and 1.68*10-7. b-c) Bar charts showing the prevalence of metastasic (b), p value = 9.21*10-3, and sarcomatoid (c), p value = 1.80*10-4 features in NF2wt/9pwt, NF2KO/9pwt, NF2wt/9p^−^, and NF2KO/9p- cases in the MSKCC cohort (N = 52, 10, 51, and 21 patients, respectively). d) Representative H&E stained images from two MSKCC cohort cases. Upper panel: NF2KO/9pwt; bottom panel: NF2KO/9p-. Images representative of the genomic background. In the latter, sarcomatoid features are readily observed. e) Kaplan–Meier survival analysis of human RCCs with and without sarcomatoid features in MSKCC (N = 16 vs N = 97 patients) (left panel), TCGA (N = 45 vs N = 743 patients) (middle panel) and TRACERx (N = 10 vs N = 91 patients) (right panel) cohorts, p values = 2.37*10-4, p value < 1*10-15 and p value = 0.016. f) Kaplan–Meier survival analysis of human RCCs with and without 9p loss features in MSKCC (N = 72 vs N = 62 patients) (left panel), TCGA (N = 140 vs N = 658 patients) (middle panel) and TRACERx (N = 57 vs N = 38 patients) (right panel) cohort, p values = 0.023, 1.21*10-8, 0.045. g-h) Bar chart showing the prevalence of sarcomatoid features in 9pwt and 9p- cases in TCGA pan-RCC dataset (N = 648 vs N = 140 tumors) (g), p value = 0.025, and TRACERx RCC dataset (N = 45 vs N = 61 tumors) (h). i-j) Bar chart showing the prevalence of stage I/II and stage III/IV features in 9pwt and 9p- cases in TCGA pan-RCC dataset (N = 628 vs N = 136 patients) (i) and TRACERx RCC dataset (N = 45 vs N = 61 patients) (j), p values = 1.56*10-8 and 9.29*10-5. k-l) Bar chart showing the prevalence of metastasis features in 9pwt and 9p- cases in TCGA pan-RCC dataset (N = 628 vs N = 136 patients) (k) and TRACERx RCC dataset (N = 45 vs N = 61 patients) (l), p values = 2.86*10-4 and 0.008. ns.: not significant, * P < 0.05, ** P < 0.01, *** P < 0.001, **** P < 0.0001 by two-sided Fisher’s exact test (b, c, g-l), log-rank (Mantel–Cox) test (a,e, f). Scale bar: 100 μm.

Metastatic progression of solid tumors is the main cause of death in patients with cancer^1^. Next-generation sequencing (NGS) studies have provided detailed annotation of the genomic landscape of metastatic cancers; however, our understanding of the role of specific genomic events in driving the emergence of clones with metastatic competencies is still elusive^1,2^. Among different tumor types, metastatic renal cell carcinoma (RCC) represents an excellent cancer model to study the role of specific genomic events in tumor progression and to functionally establish a genotype–phenotype evolutionary map^2,3^. RCCs are relatively indolent tumors that can be effectively treated with conservative strategies; however, up to a third of patients present with or progress to an aggressive form of the disease characterized by widespread systemic dissemination^4^. Understanding pathophysiological drivers leading to aggressive forms of RCC and metastatic dissemination is, for this reason, of critical importance^5^. NGS analysis of advanced RCC and phylogenetic reconstruction of tumor evolution through multiregional sequencing have identified genetic lesions and patterns associated with the emergence of metastatic lineages, including the disruption of epigenetic modulators (*SETD2*, *BAP1*), regulators of cell-cycle checkpoints (*TP53*, *CDKN2A/B*) and cell fate (*NF2*, *FAT1*), along with the presence of multiple clonal drivers and complex karyotypic features (loss of 14q and 9p)^3,6–12^ (Extended Data Fig. 1a–l and Supplementary Table 1), providing an excellent model to functionally dissect genome–phenome associations and understand whether these events are functional metastatic drivers or rather an epiphenomenon of stochastic cancer evolution^13^. We, therefore, set to generate high-throughput in vivo and ex vivo platforms of somatic mosaic genetically engineered mouse models (SM-GEMM) leveraging CRISPR–Cas9-based genome editing, to functionally capture the evolutionary patterns and clinical characteristics of metastatic RCC. This approach allowed us to explore specific genomic rearrangements and their influence on the acquisition of metastatic competencies. Genomic annotation of SM-GEMM revealed common patterns of alterations to metastatic dissemination in human and murine models, confirmed through cross-species analysis of recurrent genomic features. Our study functionally proves the role of evolutionary conserved patterns of aneuploidy, acquired through chromosomal instability (CIN), in driving malignant progression of renal cancer. We discovered that renal tumors converge on the acquisition of a ‘CIN-tolerant’ phenotype through disruption of the interferon signaling pathway. These findings provide critical insights on common evolutionary conserved paths leading to metastatic progression in otherwise indolent tumors.

## Results

### 9p loss drives acquisition of metastatic competency in RCC

**Fig. 1:**
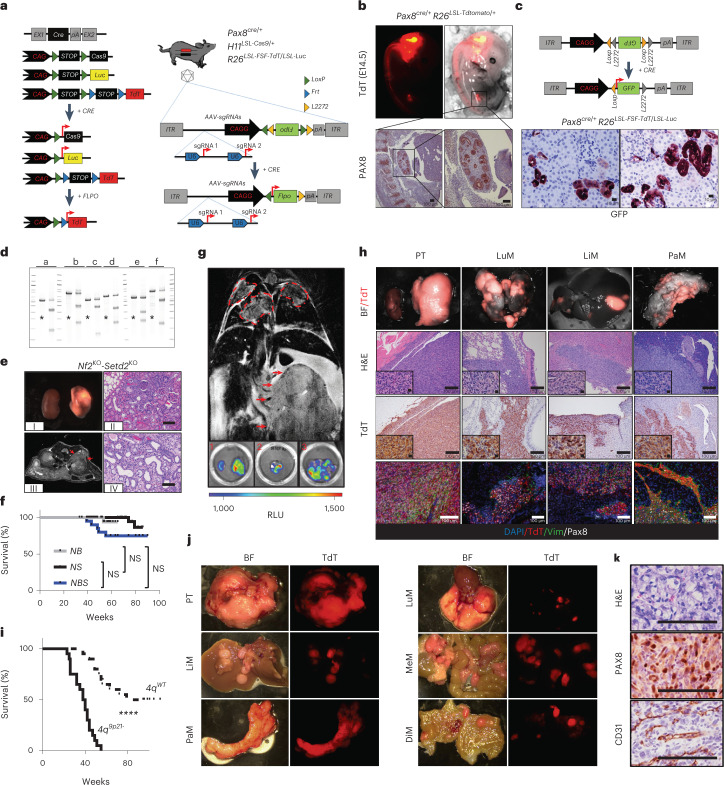
SM-GEMM of RCC. **a**, Schematic showing the SM-GEMM design. Cancer-specific loss-of-function mutations are introduced via intraparenchymal delivery of AAV particles carrying specific sgRNA combinations. **b**, Representative E14 *Pax8*^*Cre/+*^ *-R26*^*LSL-TdT/+*^ embryos. The activation of the fluorescent reporter TdT can be readily appreciated in the developing hindbrain, notochord and kidney. *n* = 5 embryos. **c**, Schematic showing the AAV-based tracing system carrying a FLEx-GFP-reported sequence. IHC analysis on representative FFPE sections stained with a GFP-specific antibody. *n* = 5 mice. **d**, T7-endonuclease assay validating sgRNA for *Trp53* (a), *Nf2* (b), *Bap1* (c), *Setd2* (d), *Cdkn2a* (e), *Cdkn2b* (f) and negative controls (*). Images representative of *n* = 3 independent experiments. **e**, Pathological characterization of murine RCC obtained through somatic mosaic knockout of *Nf2* and *Setd2*. (I) Gross specimens collected 8 months posttransduction; (II) axial T2 MRI scan displaying a small cortical lesion 8 months posttransduction; and (III) and (IV) hematoxylin and eosin (H&E)-stained sections from well-differentiated tumors collected at 6 and 8 months posttransduction, respectively. **f**, Kaplan–Meier analysis of cancer-specific survival of mice affected by Nf2KO-driven tumors. *NB*: *Nf2*^*KO*^*-Bap1*^*KO*^ (*n* = 40 mice); *NS*: *Nf2*^*KO*^*-Setd2*^*KO*^ (*n* = 20 mice); *NBS*: *Nf2*^*KO*^*-Setd2*^*KO*^-*Trp53*^*KO*^ (*n* = 24 mice). *P* = 0.23, 0.054, 0.12. **g**, Upper panel, representative coronal T2 MRI scan at 3 months posttransduction in *Nf2*^*KO*^*-Setd2*^*KO*^-*4q*^*9p21*^ mice. Red arrows, primary tumor mass; red dashed lines, lung metastasis. Bottom panels, representative luminescence scans of mouse organs. 1, primary tumor; 2, lung metastasis; 3, liver metastasis. Images representative of *n* = 2 experiments. **h**, Characterization of *Nf2*^*KO*^-driven murine tumors upon genetic targeting of the murine locus syntenic to human 9p21.3 (*4q*^*9p21*^): representative macroscopic images (top panels), H&E, IHC and IF analysis (lower panels). Images representative of *n* = 2 experiments. **i**, Kaplan–Meier analysis of cancer-specific survival of mice affected by *Vhl*^*KO*^-driven tumors with (*n* = 20 mice) or without (*n* = 20 mice) *4q*^*9p21*^ loss, *P* = 1.18 × 10^−8^. **j**,**k**, Characterization of *Vhl*^*KO*^-driven murine tumors upon genetic targeting of *4q*^*9p21*^ locus: representative macroscopic images (**j**), H&E and IHC analysis of specific clear cell RCC markers (PAX8 and CD31) are shown (**k**). PT, primary tumor; LuM, lung metastasis; LiM, liver metastasis; PaM, pancreatic metastasis; MeM, mesenteric metastasis; DiM, diaphragm metastasis. Images representative of *n* = 2 experiments. NS, not significant; ****P < 0.0001 by log-rank (Mantel–Cox) test. Scale bar, 200 μm. BF, brightfield; E, embryonic day; FFPE, formalin-fixed paraffin-embedded; IF, immunofluorescence; MRI, magnetic resonance imaging; RLU, renilla luciferase. Source data

**Fig. 2:**
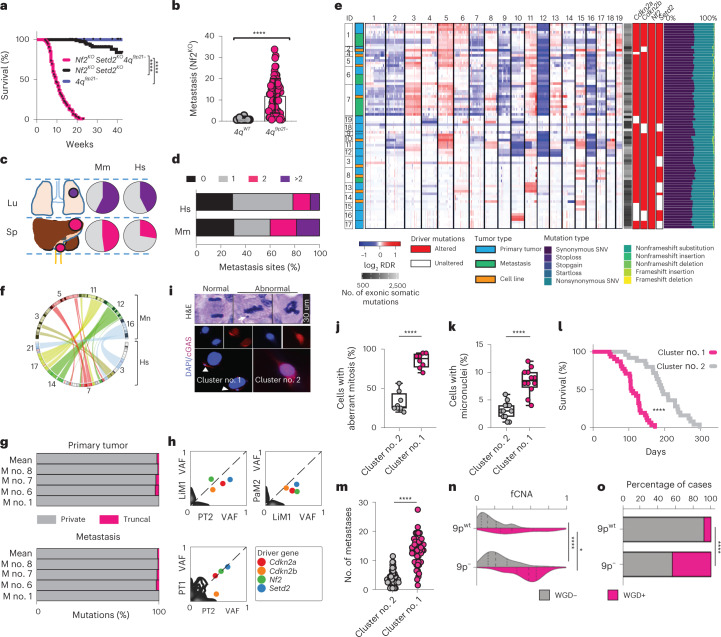
CIN is a feature of aggressive metastatic RCC. **a**, Kaplan–Meier survival analysis of *Nf2*^KO^-driven tumors with (*n* = 99 mice) and without (*n* = 84 mice) *4q*^*9p21*^-targeting sgRNAs. *P* < 1 × 10^−15^. **b**, Box and whiskers plot showing metastatic burden of *4q*^*9p21*^ (*n* = 69 mice) and *4q*^*wt*^ (*n* = 15 mice) models; data are presented as mean ± s.d., *P* = 1.16 × 10^−6^. **c**,**d**, Cross-species comparison of site-specific metastasis (**c**) and disease burden (**d**); Mm, *Mus musculus*, *n* = 79 mice; Hs, *Homo sapiens*. **e**, Summary heatmap showing WES results (*n* = 81 samples derived from 19 mice) (Supplementary Table 1). **f**, Circos plot of the human to mouse synteny map for chromosome regions significantly altered in SM-GEMM. Statistics derived from *n* = 81 samples. **g**, Bar charts showing the percentage of private and truncal somatic events at primary (upper panel) and metastatic sites (bottom panel). **h**, Density plots displaying the VAF of observed somatic mutations. **i**, Histological high-power field magnification of normal anaphase (top left) and aberrant metaphases (top right) with IFs for cGAS (red) and DAPI (blue) (middle and bottom panels). Arrows indicate micronuclei. Scale bar, 30 μm. Images representative of *n* = 3 experiments. **j**,**k**, Box and whiskers plots showing percentages of tumor cells with aberrant mitosis (**j**), data are represented as median values, minimum and maximum (26.6, 20, 56.6 for Cluster no. 2 and 70, 88 and 95 for Cluster no. 1, respectively); and with micronuclei (**k**), data are represented as median values, minimum and maximum (3, 1, 6 for Cluster no. 2 and 8.5, 4 and 12 for Cluster no. 1, respectively). *n* = 8 tumors per condition (**j**), *n* = 12 tumors per condition (**k**); *P* = 1.80 × 10^−7^ (**j**) and 1.34 × 10^−6^ (**k**). **l**,**m**, Kaplan–Meier survival analysis (**l**) and metastatic lesions count (**m**) in Cluster no. 1 and Cluster no. 2 RCC GEM models transplants; *P* = 3.08 × 10^−10^ (**l**, *n* = 57 mice) and <1 × 10^−15^ (**m**, *n* = 109 mice). **n**, Violin plot showing aneuploidy score with 9p status and WGD (9p^−^, *n* = 212 tumors; 9p^wt^, *n* = 710 tumors); *P* < 1 × 10^−15^ and *P* = 3.07 × 10^−2^. **o**, Bar chart showing the prevalence of WGD in 9p^wt^ and 9p^−^ cases in TCGA and MSKCC datasets (*n* = 922 tumors); *P* < 1 × 10^−15^. *P < 0.05, ****P < 0.0001 by log-rank (Mantel–Cox) test (**a**,**l**), two-tailed *t*-test (**b**,**j**,**k**,**m**), two-tailed Mann–Whitney test (**n**) and two-sided Fisher’s exact test (**o**). Lu, lung; M, mouse; RDR, read depth ratio; Sp, splanchnic. Source data

To investigate acquisition of metastatic potential in RCC, we engineered combinations of tissue-specific somatic knockouts of murine orthologs of the most common tumor suppressor genes (TSGs) driving RCC progression *(Vhl*, *Nf2*, *Setd2*, *Bap1* and *Trp53)*, via renal subcapsular administration of adeno-associated viral (AAV) particles carrying single-guide RNAs (sgRNAs) targeting the renal epithelium of mice expressing a tissue-specific conditional Cas9 allele and fluorescent reporters for tracing purposes (Fig. 1a–d). Combinations of these common TSGs consistently yielded indolent tumors, characterized by low penetrance, long latency and limited invasive potential with histopathological features of well-differentiated carcinomas, suggesting that somatic inactivation of the aforementioned genes is not sufficient to promote aggressive disease and metastatic spread (Fig. 1e,f). We, therefore, designed a pair of sgRNAs targeting the cell-cycle regulator genes *Cdkn2a* and *Cdkn2b* on murine chromosome 4 syntenic to human 9p21.3 (*4q*^*9p21*^), a recurrent chromosomal aberration associated with metastatic progression in patients affected by RCC^3^. Strikingly, somatic genetic manipulation of the *4q*^*9p21*^ locus in combination with *Nf2* and *Setd2* knockouts or *Vhl* and *Setd2* knockouts resulted in the emergence of rapidly fatal tumors with a prominent tendency for widespread systemic dissemination and extensive sarcomatoid differentiation (sarcomatoid Renal Cell Carcinoma, sRCC), as assessed by clinical and histopathological analysis (Figs. 1g–k and 2a,b). These features are consistent with aggressive RCC and closely mirror the patterns of metastatic dissemination of patients affected by advanced forms of the disease^14^ (Fig. 2c,d).

### Convergent genomic evolution of RCC

**Extended Data Fig. 2:**
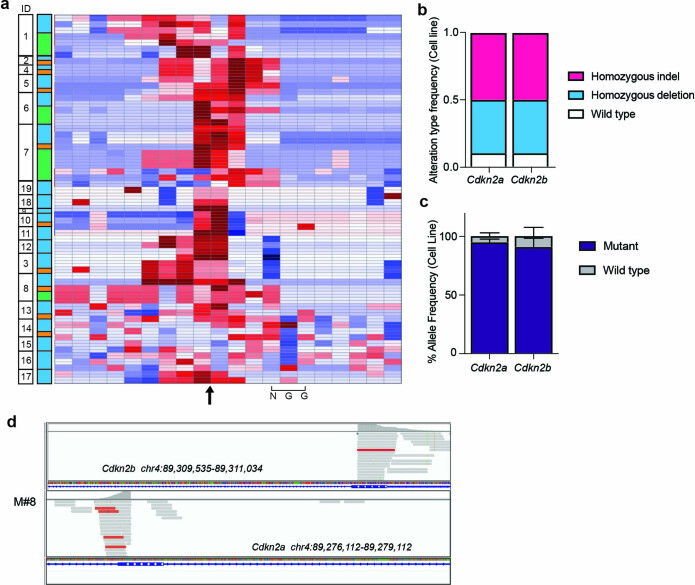
Efficiency of *in vivo* CRISPR/Cas9 genome editing in SM-GEMM of RCC. a) Heatmap showing the average Z-transformed log odds ratio across all edited genes for the likelihood of specific base alterations in any reads spanning an expected cut site. Data in figure were generated from WES analysis of primary tumor samples and matched metastatic sites (N = 81 samples across 19 mice). b-c) Bar plot displaying type (b) and allelic frequency (c) of genomic alterations in Cdkn2a and Cdkn2b loci of tumor-derived cell lines from WES data analysis, data are represented as frequencies (b) and mean values +/- SD (c) (N = 4 and 5 cell lines respectively). d) Representative IGV snapshot showing homozygous deletion of the envisaged targeted ~ 40 kb region spanning Cdkn2a and Cdkn2b genes. Source data

**Extended Data Fig. 3:**
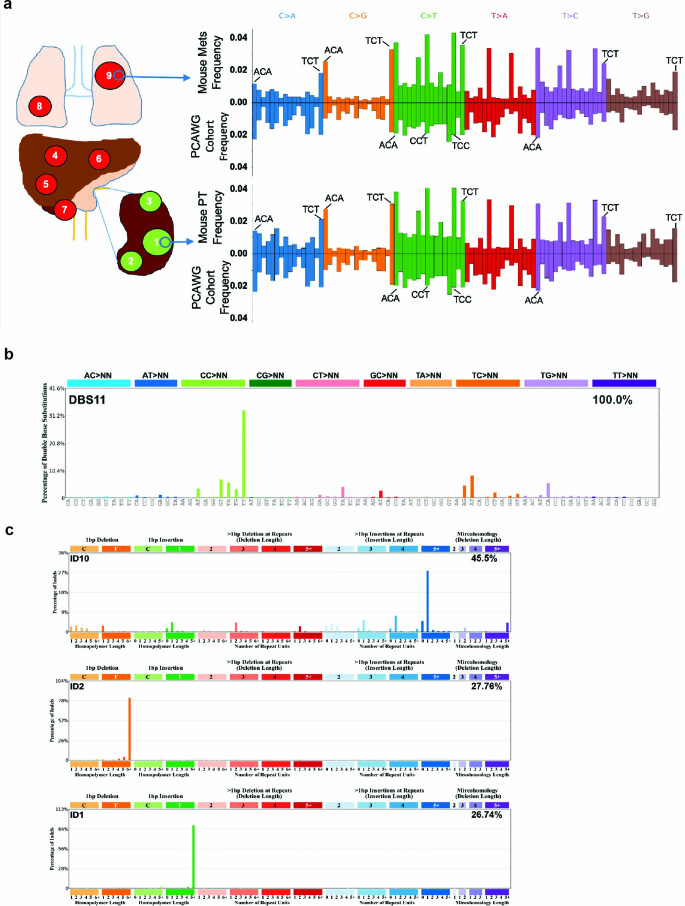
Mutational signatures of metastatic disease. a) Trinucleotide context-specific somatic SNV frequencies as detected by WGS in 2 metastatic samples (upper panel), 6 primary tumors and 2 cell line samples (lower panel) as compared to corresponding trinucleotide context-specific somatic SNV frequencies in pan-kidney tumor cohort (n = 148 tumors) analyzed by WGS. b-c) Comprehensive SM-GEMM cohort frequency plot showing percentage of double base substitutions (b) and indels (c) as calculated from WES data analysis.

**Extended Data Fig. 4:**
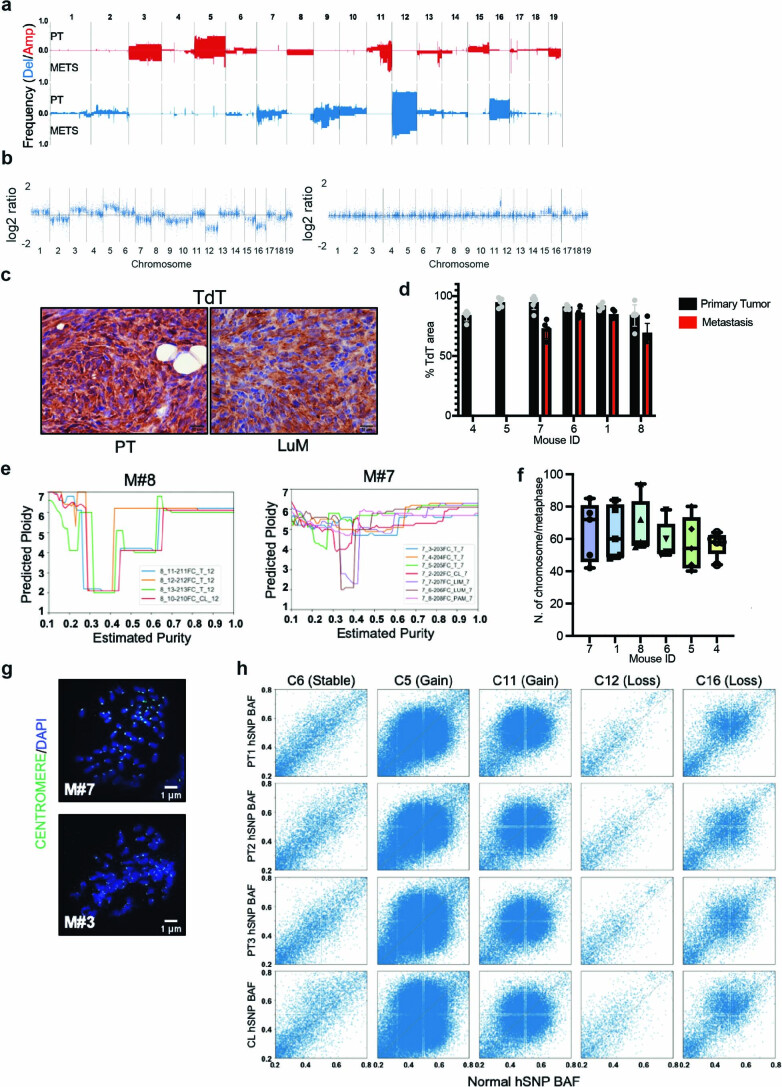
WGD and polyploidization are critical events in metastatic RCC. a) Summary of segment-level amplification or deletion frequency across murine primary tumors or metastatic lesions as determined by GISTIC2. b) Representative scatter plots of exon-level log2(Read-Depth Ratios) as calculated by CNVkit from primary tumor derived cell lines from tumor within Cluster #1 (left panel) and Cluster #2 (right panel), where different patterns of chromosomal alterations can be appreciated. c) Representative sections of TdT stained tumor tissues. Images representative of N = 2 experiments. d) Cellularity estimation of primary and metastatic samples as assessed through TdT positive cell quantification, data are presented as mean values +/- SD (N = 4 fields per tumor). e) Most probable ploidy by log posterior probability at given sample’s cellularity as predicted by Sequenza from WGS data (representative mouse #7 and #8. f) Chromosome counts in RCC SM-GEMM–derived short-term cultures. Malignant cells are characterized by prominent polyploidy, data are represented as median values, minimum, maximum (M#7: 72, 42, 85; M#1: 60, 48, 84; M#8: 58, 55, 94; M#3: 52, 50, 78; M#5: 54, 40, 80; M#4: 58, 44, 64) with boundaries at the 25th and 75th percentile (N = 5/line tested). g) Costaining of chromosomes (DAPI) and centromeres in representative nuclei of metaphase short-term cultures, established from Nf2KO-Setd2KO-Trp53KO-4q9p21 tumor-bearing mice. Images representative of N = 2 experiments. h) Comparison of primary tumor sample and matched normal B-allele frequencies (BAF) of heterozygous SNPs derived from WGS in the matched normal tissue sample (0.2 ≤ normal sample SNP BAF ≤ 0.8). The analysis was performed on chromosomes undergoing gains (5q, 11q) or losses (12q, 16q). A copy-neutral chromosome was used as control (6q). Correlation of SNP BAFs between tumor and matched normal samples. The BAFs of heterozygous SNPs suggest that WGD precedes somatic CNVs. Error bars represent the standard deviation of technical replicates. Scale bar: 100 μm. Source data

**Extended Data Fig. 5:**
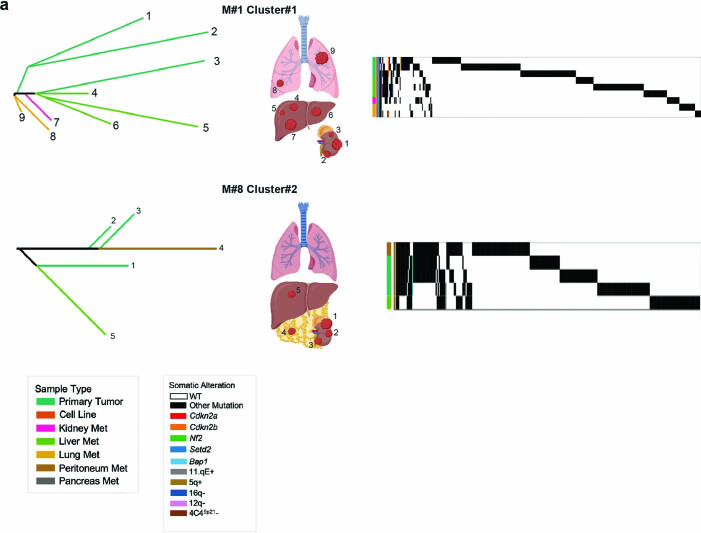
Genomic evolution of metastatic disease. a) Phylogenetic sample trees of Cluster#1 (top left) and Cluster#2 (bottom left) RCC tumor-bearing mice; different pattern of evolution can be clearly appreciated. Oncoprints of Cluster#1 (top right) and Cluster#2 (bottom right) RCC tumor-bearing mice displaying time of driver engineered and spontaneous somatic events in primary and secondary lesions. Source data

To dissect molecular drivers of aggressive murine RCC, we set to perform genomic characterization through multiregional whole-exome sequencing (WES) and, in selected cases, whole-genome sequencing (WGS) on a total of 100 samples (50 primary lesions, 21 metastatic sites, 10 tumor-derived cell lines and 19 matched healthy controls) from 19 different SM-GEMMs. We focused our genomic analysis on *Nf2*-*Setd2-4q*^*9p21*^-driven models (Supplementary Table 1). In vivo somatic mosaic engineering revealed a highly efficient in vivo editing, allowing for the detection of *4q*^*9p21*^ disruption as a consequence of homozygous indels or deletions spanning *Cdkn2a* and *Cdkn2b* genes (Extended Data Fig. 2a–d and Supplementary Table 1). Additionally, we investigated the mutational profiles of murine tumors, revealing remarkable similarities with human RCC, including a relatively low mutational burden (0.34 somatic, exonic mutations; variant allele frequency (VAF) ≥ 0.1 per Mb) and highly consistent repertoires of mutational signatures at both primary and metastatic sites (Extended Data Fig. 3a–c). Specifically, a relative prevalence of Signature 1 (C>T) consisting of spontaneous cytidine deamination is suggestive of cross-species convergent evolution in the mutational processes emerging in RCC^15^. We next performed copy number variation (CNV) analysis of primary tumors and metastatic sites; strikingly, we discovered the emergence of highly recurrent CNV events, such as loss of chromosomes 12 and 16 and gain of chromosome 5 (Fig. 2e and Extended Data Fig. 4a,b) Cross-species genomic analysis demonstrated remarkable similarities between mouse and human RCCs, as evidenced by comparative examination of syntenic genomic regions (Fig. 2f). To further characterize genomic determinants of metastatic RCC, and specifically the timing of emergence of these specific karyotypes, we inferred tumor ploidy through analysis of heterozygous single-nucleotide polymorphisms (SNPs) and identified that whole-genome duplication (WGD) events precede the emergence of specific chromosomal alterations (Extended Data Fig. 4c–h). These observations along with a minimal presence of truncal single-nucleotide variant (SNV) events are consistent with the early selection and fixation of abnormal karyotypes and the rapid expansion of clones with high fitness^3,16^ (Fig. 2g,h and Extended Data Fig. 5a).

The emergence of complex karyotypes through CIN has been uniformly associated with worse prognosis and poor response to therapy across cancer types; however, there is limited functional proof of whether specific alterations are conductive to metastatic competence or rather an epiphenomenon^1,17^. Genomic characterization of murine tumors identified two distinct genomic clusters, characterized by recurrent patterns of CNVs and a relatively unstable genome (Cluster no. 1) or few whole-chromosome alterations and inconsistent patterns of CNVs (Cluster no. 2). Cytological analysis of Cluster no. 1 and Cluster no. 2 tumors revealed, in the former, increased aberrant mitosis and presence of micronuclei resulting in the engagement of the cGAS/STING pathway through cytoplasmic DNA accumulation (Fig. 2i–k)^18^. Phenotypic analysis demonstrated that tumor explants established from Cluster no. 1 primary tumors are characterized by an aggressive clinical course with higher penetrance, shorter survival and a significant increase in metastatic burden (Fig. 2l,m). To further corroborate the association between complex karyotypes and aggressive RCC, we analyzed genomic and clinical data from The Cancer Genome Atlas (TCGA) RCC cohort, showing that *9p21* loss tumors are characterized by high fraction of copy number altered (fCNA) genome and presence of WGD (Fig. 2n,o). Altogether, these data show that the acquisition of genomic instability is pervasive in *9p21*-altered RCCs, contributing to the emergence of aggressive tumor cell populations.

### Functional heterogeneity of aggressive RCC

**Fig. 3:**
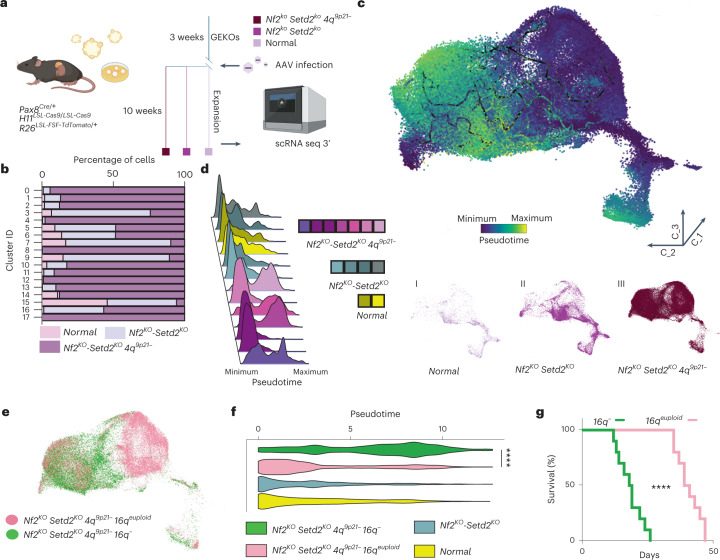
Chromosome 16q loss is permissive for the emergence of aggressive tumors. **a**, Schematic showing GEM model design for GEKOs generation (left) and experimental timeline (right) (dark purple, *Nf2*^*KO*^*-Setd2*^*KO*^*-4q*^*9p21−*^; purple, *Nf2* ^*KO*^*-Setd2* ^*KO*^; pink, empty vector). **b**, Bar graph displaying distribution of cells among 18 different clusters for the 3 different experimental groups. **c**, Three-dimensional distribution of the 87,718 GEKO-derived cells; the color scale bar is based on pseudotime values. **d**, Distribution plots of individual samples according to pseudotime values (left panel) and three-dimensional distribution along the pseudotime of the three different experimental groups (right panels). **e**, Three-dimensional distribution across the pseudotime of cells with euploid *16q* (*Nf2*^*KO*^*-Setd2*^*KO*^*-4q*^*9p21−*^*16q*^*euploid*^, pink) and with *16q*^*−*^ (*Nf2*^*KO*^*-Setd2*^*KO*^*-4q*^*9p21−*^*16q*^*−*^, green). *n* = 87,718 cells. **f**, Violin plot showing pseudotime distributions in the four different genomic groups; *P* < 1 × 10^−15^. **g**, Kaplan–Meier survival analysis of CB17SC-F SCID mice inoculated orthotopically in the kidney with SM-GEMM-derived cell lines, *16q*^−^ (*n* = 10 mice) or *16q*^*euploid*^ (*n* = 10 mice); *P* = 3.23 × 10^−6^. ****P < 0.0001 by two-tailed Mann–Whitney test (**f**) and by log-rank (Mantel–Cox) test (**g**). Source data

**Extended Data Fig. 6:**
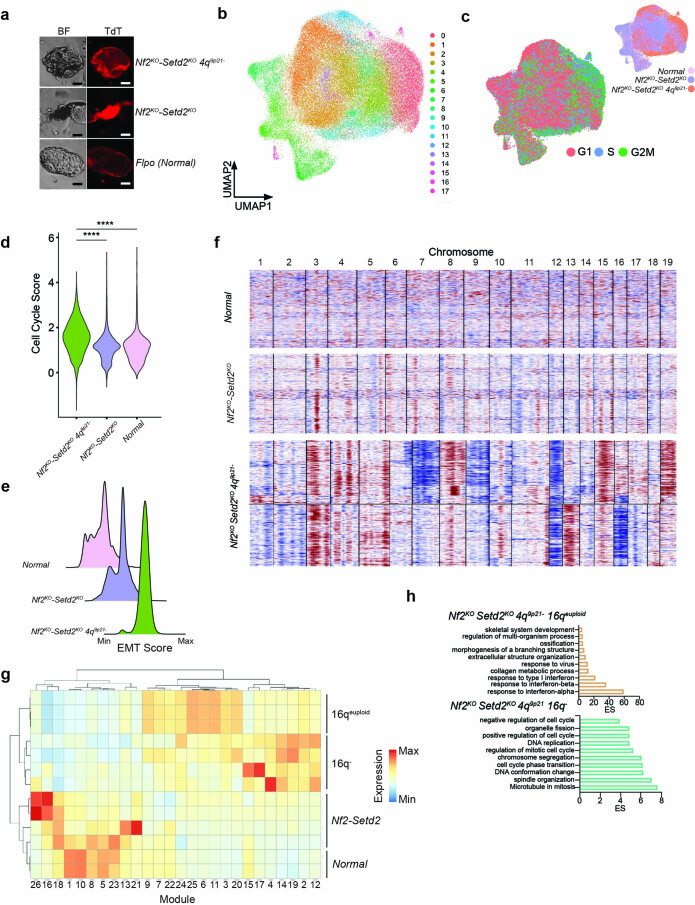
scRNA sequencing characterization of GEKOs. a) Microscopic representative pictures of GEKOs 10 weeks after AAV transduction for the three different experimental groups. Images representative of N = 3 experiments (left: brightfield; right: tdTomato). b) Bi-dimensional cluster distribution of the 87718 GEKOs cells after filtering and quality control distributed on a UMAP plot. c-d) Cell cycle status and group distribution of single GEKOs cells as calculated by Seurat (c) and violin plot of Cell cycle score values (d), p < 1*10-15, for the 3 different experimental groups. N = 87718 cells. e) Ridge plots of representing the distribution of single cells along a calculated EMT signature (EMT Score). f) Copy number heatmap of representative samples of the Normal, Nf2KO-Setd2KO and Nf2KO-Setd2KO-4q9p21 experimental groups generated by InferCNV; CIN can be appreciated in the Nf2KO-Setd2KO-4q9p21 with recurrent CNA patterns. g) Heatmap showing upregulated and downregulated modules as calculated by Moncole3 in the 4 distinct genomic groups. A clear difference among modules can be appreciated between 16qeuploid and 16q-. h) Over representation pathway analysis of top markers calculated by Seurat for the 16qeuploid (top panel) and 16q- (bottom panel) cell lines. **** P < 0.0001 by Mann-Whitney test (d). Source data

To dissect molecular pathways involved in RCC progression following *4q*^*9p21*^ loss, we generated genetically engineered kidney organoids (GEKOs) carrying somatic knockouts of *Nf2* and *Setd2* TSGs along with the inactivation of *Cdkn2a/b* on chromosome *4q*^*9p21*^ and performed single-cell RNA-sequencing (scRNA-seq) analysis, aiming to provide a dynamic multi-dimensional landscape of 9p deletion in RCC evolution (Fig. 3a and Extended Data Fig. 6a). After quality filtering, 87,718 cells were retrieved from 13 samples clustering among 18 different subtypes. Computational deconvolution of inferred trajectories of GEKO cells revealed multiple routes of transcriptomic heterogeneity upon loss of *4q*^*9p21*^ across two independent algorithms^19,20^ (Fig. 3b,c, Extended Data Fig. 6b and Methods). In spite of generally low levels of genetic heterogeneity and early selection of malignant clones with high fitness and complex karyotypes, as observed from genomic analysis, scRNA-seq data suggest that CIN favors the emergence of transcriptomic variability in the context of aggressive organoid models (*Nf2*^*KO*^*-Setd2*^*KO*^-*4q*^*9p21*−^) and an overall increase of transcriptomic heterogeneity when compared with wild-type or *Nf2*^*KO*^*-Setd2*^*KO*^ organoids. Furthermore, *4q*^*9p21*^ organoids displayed a significant enrichment for genes involved in cell-cycle progression, with a higher fraction of cells harboring transcriptomic features of S or G2/M phases along with markers of mesenchymal plasticity and sarcomatoid differentiation. These evidences support our previous observations in SM-GEMMs and are in line with patient-derived data^21^ (Extended Data Fig. 6c–e).

**Fig. 4:**
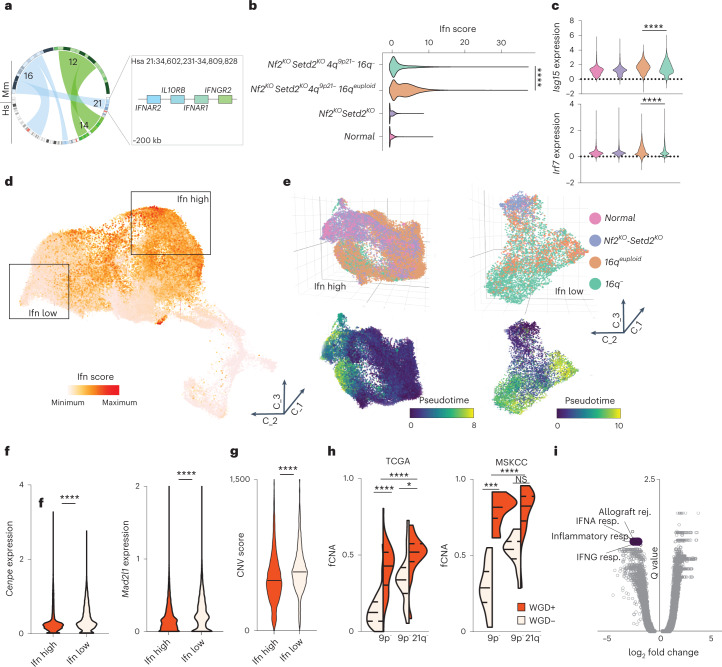
Interferon signaling suppression drives expansion of aneuploid RCC clones. **a**, Circos plots of the human to mouse synteny map for chromosome regions significantly lost in SM-GEMM tumor-bearing mice, generated by the SynCircos function of Synteny Portal. Magnification of the human chromosome 21 region shows the genomic location and coordinates of the IFNR cluster. **b**, Violin plot displaying interferon (Ifn) score calculated for four different groups clustered by genomic data (*P* < 1 × 10^−15^, *n* = 87,718 cells). **c**, Violin plots displaying expression values of *Isg15* (top) and *Irf7* (bottom) calculated for four different groups clustered by genomic data (*P* < 1 × 10^−15^, n = 87,718 cells). **d**, Three-dimensional distribution of the Ifn score values for all the cells. **e**, Three-dimensional representation of two subpopulations with high values of Ifn score (left panel) and low values of Ifn score (right panel), displaying the distribution in the four different genomic groups and pseudotime values. *n* = 87,718 cells. **f**, Expression values of two genes involved in chromosome stability and mitotic checkpoint in the Ifn low and Ifn high groups; *P*  < 1 × 10^−15^. *n* = 37,624 cells. **g**, Violin plot displaying the CNV score in the ‘Ifn high’ and ‘Ifn low’ groups; *P* < 1 × 10^−15^. *n* = 37,624 cells. **h**, Violin plot displaying fCNA values across different tumors with 9p^−^ or 9p^−^ and 21q^−^, with or without WGD, in two different cohorts: TCGA-KIPAN (left panel), *P* = 2.76 × 10^−7^, 1.67 × 10^−2^ and 1.46 × 10^−5^; MSKCC (right panel), *P* = 1.76 × 10^−4^ and 5.77 × 10^−5^. *n* = 922 tumors. **i**, Volcano plot showing top upregulated and downregulated pathways, comparing 9p^−^ and 21q^−^ tumors versus 9p^−^ tumors in the TCGA-KIPAN transcriptomic dataset. *n* = 788 tumors. *P < 0.05, **P < 0.01, ***P < 0.001, ****P < 0.0001 by two-tailed Mann–Whitney test (**b**,**f**–**h**). Rej., rejection; resp., response; TGCA-KIPAN, TCGA pan-kidney. Source data

**Fig. 5:**
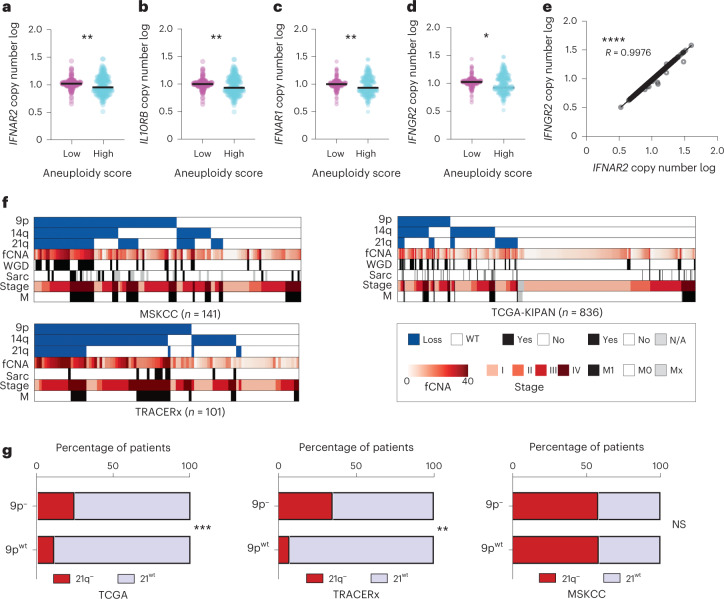
CIN is associated with interferon signaling suppression in RCC. **a**, Dot plot showing copy number log values of the *IFNAR2* gene across human cell lines derived from nonhematological malignancies as calculated from the Cancer Cell Line Encyclopedia (CCLE). Cell lines were divided based on their aneuploidy score; *P* = 0.0099. **b**, Dot plot showing copy number log values of the *IL10RB* gene, across the same cell lines as a. *P* = 0.0099, *n* = 789 cell lines. **c**, Dot plot showing copy number log values of the *IFNAR1* gene, across the same cell lines as a. *P* = 0.00992, *n* = 789 cell lines. **d**, Dot plot showing copy number log values of the *IFNGR2* gene, across the same cell lines as a. *P* = 0.015, *n* = 789 cell lines. **e**, Scatter dot plot copy number log values of two IFNR genes located on the specific deleted chromosome 21 region; *P* < 1 × 10^−15^. *n* = 789 cell lines. **f**, Heatmap displaying the clinical, histological and genomic annotation of specific features across MSKCC RCC cohort (upper left panel), TRACERx RCC cohort (bottom left panel) and TCGA-KIPAN cohort (upper right panel). **g**, Bar plot showing co-occurrence of 21q loss and 9p loss in the three different clinical cohorts; from left to right, *P* = 1.04 × 10^−4^ and 0.0016. From left to right, *n* = 788, 101 and 134 tumors. *P < 0.05; **P < 0.01; ***P < 0.001; ****P < 0.0001 by two-sided Mann–Whitney test (**a**–**d**), Pearson correlation (**e**) and two-sided Fisher exact chi-squared test (**g**). N/A, not applicable; WT, wild type. Source data

Analysis of single-cell trajectories revealed two major subclasses within the *4q*^*9p21*^ experimental group, with evolutionary divergence as a measure of the inferred distance from the routes’ origin (Fig. 3d). Cross-platform annotation of structural variants, as inferred from scRNA-seq on organoids, identified loss of murine chromosome 16 (*16q*^*−*^) as a genomic determinant of malignant progression and molecular divergence, confirming multiregional WES data on SM-GEMMs (Extended Data Fig. 6f). Single-cell transcriptomic analysis demonstrated that cells acquiring spontaneous loss of chromosome 16 displayed increased distance from the origin of the route, suggesting this genomic group to be the evolutionary endpoint in murine RCC (Fig. 3e,f). These observations prompted us to hypothesize that if the loss of *4q*^*9p21*^ is permissive for the emergence of clones with CIN, *16q* loss might promote tolerance to aneuploidy and ultimately being permissive to the expansion of clones with complex karyotypes. To test this hypothesis, we performed in vivo functional assays showing that transplants generated from short-term passaged *16q*^*−*^ clones exhibit a more aggressive behavior and result in reduced survival when compared with *16q*^*euploid*^ isogenic transplants (Fig. 3g), thus confirming that *16q*^*−*^ is a functional driver of cancer cell fitness and aggressive biological features in renal cancer. Remarkably, cross-species synteny analysis displayed a high level of homology between murine chromosome 16 and human chromosome 21, including a conserved ~200-kilobase genomic region harboring the interferon receptor (IFNR) cluster genes shown to be involved in type I, II and III interferon response (*IFNAR1*, *IL10RB*, *IFNAR2*, *IFNGR2*) (Fig. 4a). Accordingly, single-cell transcriptomic analysis confirmed that *16q*^*−*^ populations were characterized by a significant suppression of the interferon signaling response when compared with *16*^*euploid*^ cells (*P* < 0.0001), together with activated programs involved in the mitotic checkpoint and regulation of cell-cycle progression (Fig. 4b–f and Extended Data Fig. 6g–i). These evidences therefore suggest that the disengagement of the interferon response in the context of aneuploidy is permissive for the expansions of aggressive cancer cells and contributes to tumor heterogeneity and functional clonal diversification (Fig. 4g). Exploiting publicly available databases of human cancer cell lines across multiple solid tumor subtypes (Cancer Cell Line Encyclopedia, CCLE) and cohorts of patients with RCC with pathological and genomic annotations (TCGA; Tracking renal cancer evolution through therapy, TRACERx; Memorial Sloan Kettering Metastasis, MSK-Met), we confirmed a significant association between IFNR cluster loss and aneuploidy, leveraging metrics of aneuploidy score as WGD and fCNA genome. Analysis of multiple datasets and integration of human and mouse RCC data showed an inverse correlation between interferon signaling and CIN (Figs. 4h,i and 5a–g and Supplementary Table 1). Thus, RCCs with high levels of CIN demonstrate selective evolutionary pressure towards the suppression of the interferon response pathway through genetic loss of the IFNR cluster on chromosome 21.

### IFNR cluster is a gatekeeper of RCC progression

**Fig. 6:**
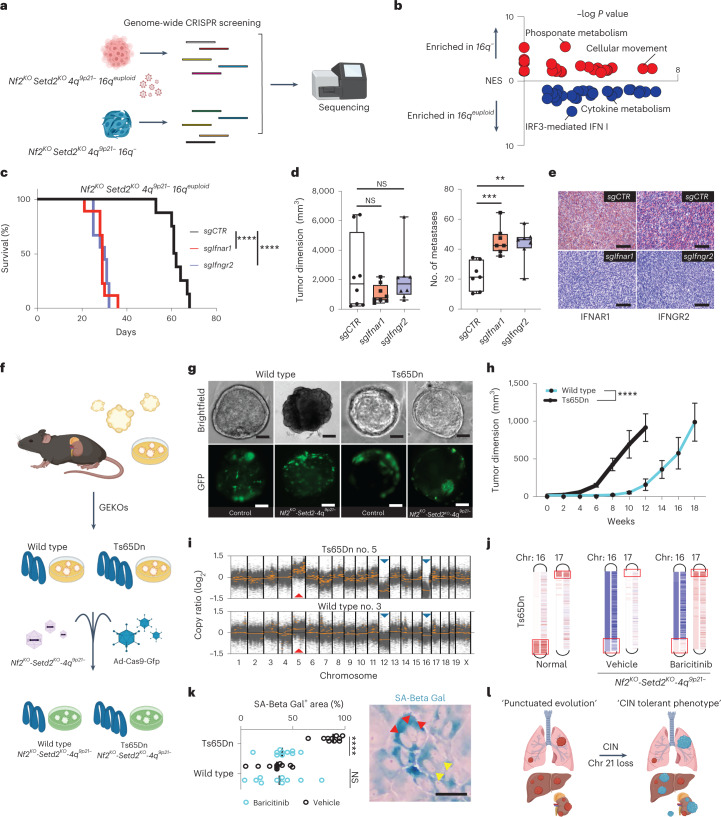
IFNR drives a senescence response that limits RCC progression. **a**, In vitro CRISPR screening schematic. **b**, Volcano plot showing enriched pathways in *16q*^*−*^ and *16q*^*euploid*^ cell lines using as input the top ranked 2,000 TSGs. *n* = 60 differentially expressed pathways. **c**, Survival curve of *16q*^*euploid*^ tumor-bearing mice with knockout of either *Ifnar1* or *Ifngr2*; *P* = 3.44 × 10^−5^ and 4.20 × 10^−5^. *n* = 26 mice. **d**,**e**, Tumor dimensions and number of metastases; data are represented as median values, minimum and maximum (*sgCTR*: 1,702.5, 198, 6,394; *sgIfnar1*: 750, 405, 2,176; *sgIfngr2*: 1,702.5, 607, 6,250 for tumor dimensions; *n* = 9 tumors per group; and *sgCTR*: 21, 10, 34; *sgIfnar1*: 42, 35, 64; *sgIfngr2*: 46, 20, 57 for number of metastases; *n* = 8 tumors per group) (**d**); and IHC of IFNAR1 and IFNGR2 in primary tumors (**e**). *P*  = 6.33 × 10^−4^, 2.72 × 10^−3^, 0.17, 0.63. Scale bar, 100 μm. **f**, Schematic of the experimental design and GEKO generation for the Ts65Dn model. **g**, Microscopic images of wild-type (top left) and Ts65Dn (top right) GEKOs coinfected with Ad-Cas9-GFP with or without the AAV-*Nf2*^*KO*^*-Setd2*^*KO*^*- 4q*^*9p21−*^. Scale bar, 30 μm. Images representative of *n* = 2 experiments. **h**, Growth curve of transformed wild-type and Ts65Dn GEKOs transplanted subcutaneously; data are presented as mean ± s.d. (wild type, *n* = 5 tumors; Ts65Dn, *n* = 5 tumors), *P* = 3.28 × 10^−9^. **i**, Scatter plots of GEKO wild-type- and Ts65Dn-derived tumors; red arrows, amplifications; blue arrows, deletions. **j**, Chromosome 16 and 17 diagrams showing regions of amplification and deletion; from left to right: normal tissue from Ts65Dn versus normal tissue from wild-type mouse; CRISPR-induced tumor from Ts65Dn treated with vehicle versus normal tissue from Ts65Dn; CRISPR-induced tumor from Ts65Dn treated with baricitinib versus normal tissue from Ts65Dn. Boxes represent the genomic region affected with partial trisomy in the Ts65Dn model. **k**, Quantification (left) and representative picture (right) of GEKTCs derived from wild-type and Ts65dn mice, treated with vehicle or baricitinib. *n* = 10 fields per condition, *P* = 2.10 × 10^−8^. Arrows indicate the presence of multiple nuclei in senescent cells. Scale bar, 30 μm. **l**, Schematic proposing loss of chromosome 21 as a cell-autonomous mechanism to CIN tolerance and evolution of advanced RCC. **P < 0.01, ***P < 0.001, ****P < 0.0001 by log-rank (Mantel–Cox) test, (**c**) two-way ANOVA (**h**) and two-tailed Student’s *t*-test (**d**,**k**). SA-Beta-Gal, beta-galactosidase. Source data

**Extended Data Fig. 7:**
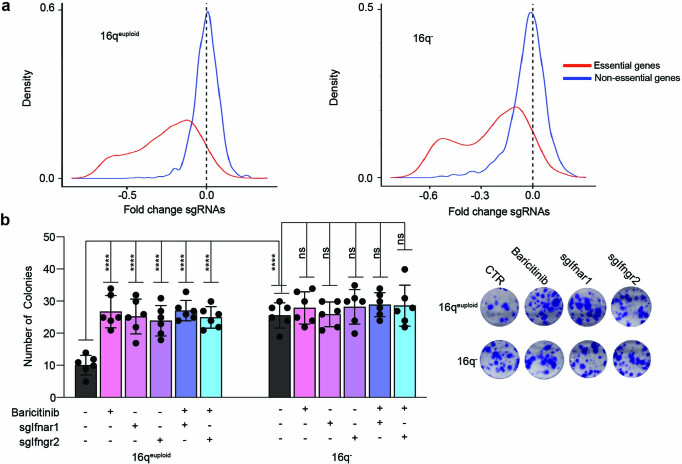
IFNRs are tumor suppressive in a cell autonomous manner. a) Genome-Wide CRISPR Screen quality control via fold change separation curves generated using a previously curated list of known essential and non-essential genes. Comparison of the foldchange of guide level abundance at 20 doublings to the reference timepoint reveals significant drop-out in the essential genes and minimal drop out in the non-essential population, indicating no change from the reference population. b) Colony forming unity assay showing number of colonies after Ifnra1 or Ifngr2 knockouts with or without Baricitinib treatment compared to parental untreated cells (left panel) and representative images of the experiment (right panel), data are presented as mean values +/- SD (N = 6 tumors per each condition), p values = 4.73*10-5, 0.0012, 5.17*10-4, 1.25*10-5, 1.47*10-4. **** P < 0.0001 by two-way Anova with Tukey’s multiple comparison (b). Source data

**Extended Data Fig. 8:**
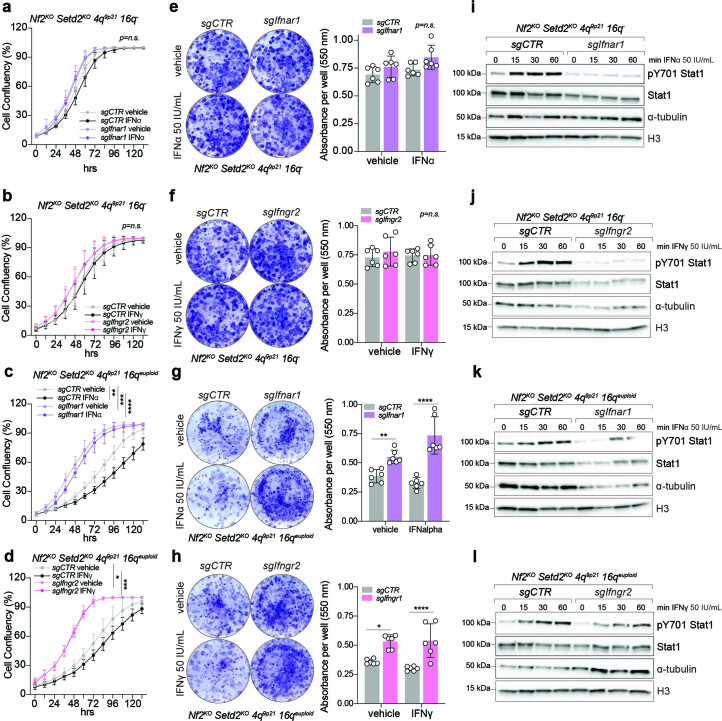
IFNRs loss protects RCC from deleterious effects of IFN type I and II treatment on cell proliferation and survival. a-d) Growth curves of 16q loss and 16q euploid RCC lines with or without knockout of Ifnar1 or Ifngr2 treated with mouse IFN-gamma, IFN-alpha or untreated. A beneficial effect of interferon receptor loss can be appreciated when 16q euploid cells are treated with IFN-alpha or IFN-gamma, data are presented as mean values +/- SD (N = 8 measurements of replicates per each time point), p values = 0.012, 0.0002 and 4.29*10-9 (c), p values 0.035 and 0.007 (d). e-h) Representative colony pictures (left) and relative quantification bar graphs (right) of 16q loss and 16q euploid RCC lines with or without knockout of Ifnar1 or Ifngr2 treated with mouse IFN-gamma, IFN-alpha or untreated. Loss of either Ifngr2 or Ifnar1 resulted in a beneficial long-term proliferative advantage only in 16q euploid RCC lines, data are presented as mean values +/- SD (N = 6 measurements of replicates per each condition), p values = 0.026 and 4.80*10-9 (g), p values = 0.018 and 3.28*10-8 (h). i-l) Western blots showing evidence of reduced STAT1 phosphorilation under interferon administration in cell lines knocked-out for either Ifngr2 or Ifnar1. Images representative of N = 3 independent experiments. * P < 0.05; ** P < 0.01; ***P < 0.001; **** P < 0.0001 by two-way ANOVA with multiple t-test (a-h). Source data

**Extended Data Fig. 9:**
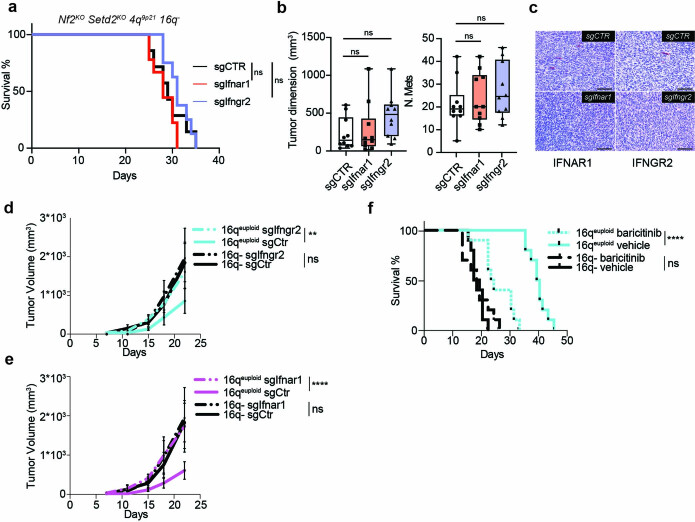
Chromosome *21q* loss and IFNR loss confer a pro-tumorigenic and pro-metastatic phenotype in RCC. a) Survival curves of 16q- tumor bearing mice with either knockout of Ifnar1, Ifngr2 or none (N = 10 mice per each condition). p value = 0.15 b-c) Clinicopathological assessment of tumor dimension and number of metastasis, data are represented as median values, minimum, maximum (sgCTR: 135.75, 32, 600; sgIfnar1: 144, 13.5, 1080; sgIfngr2: 477.5, 87.5, 1080 for Tumor dimension and sgCTR: 19, 5, 42; sgIfnar1: 20, 10, 42; sgIfngr2: 24.5, 12, 46 for number of metastasis) with boundaries at the 25th and 75th percentile (b) and immunohistochemical staining of IFNAR1 and IFNGR2 in primary tumors at endpoint (c) for 16q- tumor bearing mice. (N = 10 tumors per each condition) d-e) Growth curves of 16qeuploid and 16q- cell lines upon knock-out of Ifngr2 (N = 5 tumors per condition, d), p value = 0.065, and Ifnar1 (N = 5 tumors per condition, e). p = 5.84*10-6 after subcutaneous transplantation in NOD-SCID mice, data are presented as mean values +/- SD. f) Survival curves of 16qeuploid and 16q- tumor bearing mice treated (N = 10 mice per each condition) or not (N = 10 mice) with Baricitinib, p = 5.08*10-6. ** P < 0.01; **** P < 0.0001 by two-way Anova (b,d,e) and log-rank (Mantel–Cox) test (a,f). Source data

**Extended Data Fig. 10:**
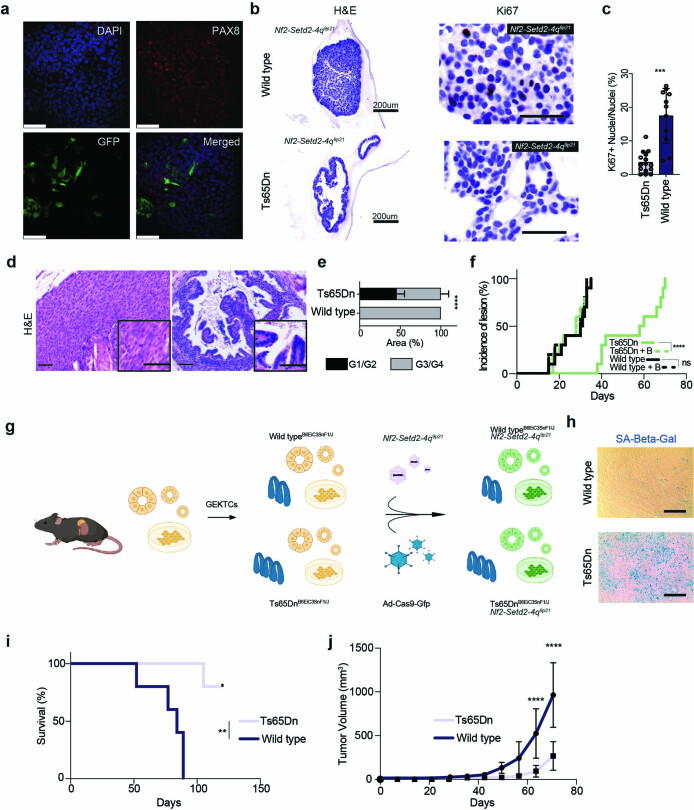
An extra copy of the IFNR cluster is sufficient to restrains malignant transformation in RCC. a) Confocal images of a GEKO infected with Ad-Cas9-GFP. The positive staining with the renal marker PAX8 confirmed a tubular origin of this organoids; GFP staining confirmed the successful transduction. Scale bar: 100μm b-c) Representative histopathological images of “Wild type” and “Ts65Dn” GEKOs cotransduced with sgRNAs-carrying AAV and Ad-Cas9-GFP stained for the proliferation marker Ki67 (b) and relative quantification (c), data are presented as mean values +/- SD, p value = 3.17*10-6. Scale bar: 100μm (N = 15 for “Ts65Dn and N = 11 for “Wild type”) Images representative of N = 2 experiments. d-e) Representative H&E images showing sarcomatoid (left panel) and tubule-papillary like (right panel) morphology for “Wild type” and “Ts65Dn” GEKO-derived tumors respectively (e); quantification of “Ts65Dn” and “Wild type” GEKO-derived tumors with histological low grade (G1/G2) and high grade (G3/G4), data are presented as mean values +/- SD (N = 25 fields per each condition, p < 1*10-15) (e). Scale bar: 100μm f) Incidence curves of tumor bearing mice transplanted with “Ts65Dn” or “Wild type” GEKOs and treated with Vehicle or Baricitinib, p value = 4.47*10-6. g) Schematic showing the generation of GEKTCs. h) Representative images of “Wild type” and “Ts65Dn” GEKTCs stained for SA-Beta-Gal. Images representative of N = 3 experiments. I-j) Survival curves of “Ts65Dn” (N = 5 mice) and “Wild type” (N = 5 mice) GEKTC cell lines transplanted in immunocompromised mice and respective tumor growth curve, data are presented as mean values +/- SD, p values = 0.0026, 1.49*10-5 and 2.74*10-11 (j). Scale bar: 100μm. ** P < 0.01, *** P < 0.001, **** P < 0.0001 by student-T test (c) Chi test (e) log-rank (Mentel-Cox) test (f-i) and two-way ANOVA (j). Source data

Orthogonal validation of the role of IFNR cluster loss in promoting tolerance to CIN was further provided through a functional genomic approach leveraging genome-wide CRISPR screens in *16q*^*−*^ and *16q*^*euploid*^ isogenic lines. Specifically, deconvolution of enriched sgRNAs coupled with Enrichment Pathway Analysis of gene targets confirmed a potent cell-autonomous tumor suppressive role for the interferon signaling pathway in renal cancer progression and a selective pressure to suppress the interferon response in *16q*^*euploid*^ cells (Fig. 6a,b, Extended Data Fig. 7a and Supplementary Table 1). To confirm CRISPR screens data and to clarify the functional effects of genes belonging to the IFNR cluster, we designed sgRNA targeting *Ifnar1* and *Ifngr2*. Single-gene knockout of *Ifnar1* and *Ifngr2*, pharmacological pathway inhibition with JAK1/2 inhibitor (baricitinib) and treatment with exogenous interferon-α (IFN-α) and -γ (IFN-γ) confirmed the tumor suppressive role of IFNR signaling in in vitro assays (Extended Data Figs. 7b and 8a–h); at the molecular level, genetic manipulation of the pathway resulted, as expected, in a significant downmodulation of STAT1 phosphorylation (Extended Data Fig. 8i–l). Similarly, in vivo transplantation studies demonstrated that genetic knockouts of *Ifnar1* or *Ifngr2* and inhibition of the JAK/STAT signaling pathway confer a pro-tumorigenic and pro-metastatic phenotype in the context of euploid copies of chromosome 16 (Fig. 6c–e and Extended Data Fig. 9a–f). These experimental evidences suggest a cell-autonomous role of the loss of the syntenic region on murine *16q* and human *21q* in tolerating the deleterious effects of interferons on the survival of cells under mitotic stress, establishing a putative causal interaction between the IFNR pathway, through JAK/STAT signaling, and proliferation of cells with CIN^22^. To provide a comprehensive overview of the functional role of *16q* loss and IFNR in RCC progression, we designed gain-of-function studies in renal organoids and normal renal tubular cell lines established from a murine model of Down syndrome with a partial trisomy of chromosome 16 spanning the IFNR cluster (Ts65Dn)^23^. Through genomic engineering of wild-type and Ts65Dn GEKOs, we introduced somatic knockouts of *Nf2* and *Setd2* TSGs and genomic disruption of chromosome *4q*^*9p21*^ via cotransduction of AAV and adenoviral particles carrying an in-frame Cas9-GFP cassette (Ad-Cas9-GFP) (Fig. 6f,g). Transplantation experiments confirmed a dosage-dependent negative effect of interferon signaling on tumor initiation and progression (Fig. 6h and Extended Data Fig. 10a–e). WES analysis of 13 cases of *Nf2*^*KO*^*-Setd2*^*KO*^*-4q*^*9p21*^ engineered wild-type- and Ts65Dn-GEKO-derived primary tumors collected at terminal stage revealed that, despite pre-existing genomic abnormalities, RCC evolution converges towards recurrent patterns of aneuploidy (gain of chromosome *5q*, losses of chromosomes *12q* and *16q*), but, more importantly, these data showed that tumor development is consistently associated with the loss of both *16q* and the engineered extra copy of chromosome *16q*. (Fig. 6i). Remarkably, chronic pharmacological suppression of the JAK-STAT signaling pathway rescued this phenotype, with tumors derived from engineered Ts65Dn organoids retaining the artificial chromosome (Fig. 6j and Extended Data Fig. 10f). These data corroborate the critical role of interferon signaling and IFNR cluster dosage in renal tumorigenesis. Further experimental evidences demonstrated that an extra copy of the IFNR cluster is sufficient to dramatically impair tumorigenesis in vivo and proliferation in vitro in SM-GEMM-derived kidney tubular cells, through the activation of a potent senescence response, which is fully rescued by pharmacological inhibition of the IFNR pathway leveraging the JAK inhibitor baricitinib (Fig. 6k and Extended Data Fig. 10g–j).

## Discussion

Altogether, we established functional proof of the central role of *9p* loss in determining patterns of metastatic disease. Despite other GEM models of renal cancer have been previosuly generated^24^, in the present study, by engineering *9p21* loss in vivo, we generated immune-competent somatic mosaic models of aggressive and metastatic RCC. We thus demonstrated the critical role of specific genomic events in triggering CIN and promoting the rapid expansion of aggressive subpopulations with prominent metastatic behavior^3,25–27^.

WES and WGS analyses provide insights into the modalities of genetic evolution in *9p* loss-driven tumors, revealing early emergence and rapid selection of clones defined by WGD, CIN and highly conserved patterns of aneuploidy. These features are in line with a model of punctuated equilibrium, where bursts of macroevolutionary events drive rapid clonal sweeps and the selection of cells with high fitness^28^. Interestingly, the proposed model informs on the existence of convergent evolutionary trajectories^29^, as evidenced by cross-species genotype–phenotype analysis, and suggests that, providing there are the appropriate initiating oncogenic drivers, the evolutionary bottlenecks shaping the cancer genome are consistent across species. This work is in line with recent papers demonstrating convergent evolutionary trajectories in murine and human pancreatic cancers, where the spontaneous loss of *CDKN2A/B*, *TP53* and *SMAD4* represents a constrained route to malignant progression^30,31^.

Analysis of scRNA-seq data showed heterogenous transcriptomic dynamics upon loss of *9p21*, unlocking an increase in the number of cell states and therefore a higher degree of tumor entropy. More importantly, this study reveals a highly conserved and critical tumor suppressive role of the interferon signaling pathway in the progression to advanced and metastatic RCC, particularly in the context of tumors with high CIN^32^ (Fig. 6l). Our findings are in line with clinical evidences showing that an increase in gene dosage at the IFNR cluster locus in patients with Down syndrome is associated with a decreased lifelong risk of developing solid tumors at the expenses of a pro-senescent cellular phenotype and a proinflammatory milieu, resulting in a higher risk of incidence of systemic inflammatory and autoimmune diseases^33–36^. The loss of type I interferon signaling has been observed upon malignant progression to metastatic dissemination and as a mechanism of immune-evasion, particularly as an adaptive response to immune checkpoint blockade in malignant melanoma and epithelial cancers, through loss of the type I interferon ligands cluster on chromosome *9p* or through mutations of *JAK1/2* (refs. ^37–39^). Here, we provide functional proof of the pivotal role of the loss of the IFNR cluster on *21q* in the progression of renal cancers and the rationale for a potential role in other tumor types. Notably, when compared with the loss of the type I ligands cluster on *9p* (ref. ^37^), *21q* loss drives suppression of both type I and II receptors, ultimately converging on STAT1 reduced activation. These evidences suggest that both type I and II responses are critical tumor suppressor pathways in RCC, particularly as an adoptive response to CIN. Further studies are therefore required to dissect tissue-specific and cancer-specific dependencies. Concluding, our work supports convergent evolutionary patterns leading to metastatic dissemination in different genomic backgrounds, suggesting that metastatic and aggressive tumor progression can be largely anticipated through the analysis of specific drivers.

## Methods

### Animal models

The Pax8^Cre^ strain was generated by Dr. Meinrad Busslinger and obtained through the Jackson Laboratory, Stock no. 028196 (ref. ^40^). The H11^LSL-Cas9^ strain was generated by Dr. Monte M. Winslow and obtained through the Jackson Laboratory, Stock no. 027632 (ref. ^41^). The Rosa26^LSL-TdT^ was generated in Dr. Hongkui Zeng’s laboratory and obtained through the Jackson Laboratory, Stock no. 007908 (ref. ^42^). The Rosa26^fsf-lsl-TdTomato^ was generated in Hongkui Zeng’s laboratory and obtained through the Jackson Laboratory, Stock no. 021875 (ref. ^43^). Rosa26^LSL-Luc^ mice were generated by Dr. William G. Kaelin and obtained through the Jackson Laboratory, Stock no. 034320 (ref. ^44^). The Ts65Dn strain was generated by Dr. Muriel T. Davisson and obtained through the Jackson Laboratory, Stock no. 001924 (ref. ^23^). Strains were kept in a mixed C57BL/6 and 129Sv/Jae background, except for the Ts65Dn which was kept in B6EiC3Sn background. Embryo collection was performed at embryonic day 14. CB17SC-F SCID mice were purchased from Taconic. All animal studies and procedures were approved by the University of Texas MD Anderson Cancer Center (UTMDACC) Institutional Animal Care and Use Committee. All experiments conformed to the relevant regulatory standards and were overseen by the institutional review board. Maximal tumor burden was not exceeded according to the institutional review board guidelines: for orthotopic tumors, mice were euthanized upon symptoms of distress; for subcutaneous transplantations, maximal tumor burden was 2 cm^3^. No sex bias was introduced during the generation of experimental cohorts. Mice were kept in a 12-h light/12-h dark cycle as commonly used, and housed at 18–23 °C with humidity of 50–60%.

### Animal procedures

#### Orthotopic kidney injection

First, 10^10^ AAV particles were resuspended in OPTI-MEM (Gibco) and Matrigel matrix (Corning) (2:1 dilution). Six- to nine-week-old mice were shaved and anesthetized using isoflurane (Henry Schein Animal Health). Analgesia was achieved with buprenorphine slow release (0.1 mg kg^−1^ two times per day) (Par Parmaceutical) via subcutaneous injection, and shaved skin was disinfected with 70% ethanol and betadine (Dynarex). A 1-cm incision was performed on the left flank through the skin/subcutaneous and muscular/peritoneal layers. The left kidney was exposed and 20 μl of viral resuspension was introduced by subcapsular injection. The kidney was carefully repositioned into the abdominal cavity, and muscular/peritoneal planes were closed individually by absorbable sutures. The skin/subcutaneous planes were closed using metal clips. Mice were monitored daily for the first 3 d, and then twice per week.

#### Subcutaneous transplantation

Tumor cells, GEKO-derived single-cell suspensions and genetically engineered kidney tubular cell (GEKTC) single-cell suspensions were resuspended in OPTI-MEM (Gibco) and Matrigel (Corning) (2:1 dilution) at a density of 2,000 cells per μl, and 100 μl of cell suspensions were injected subcutaneously into the flanks of 4–6-week-old CB17SC-F SCID female mice.

#### Treatments

Baricitinib treatment (Selleckchem, INCB028050) started the day after subcutaneous transplantation of GEKOs and GEKTCs and was administered via oral gavage at a concentration of 10 mg kg^−1^ daily until euthanasia.

#### Euthanasia, necropsy and tissue collection

Mice were euthanized by exposure to CO_2_ followed by cervical dislocation. A necropsy form was filled in with mouse information, tumor size and weight, infiltrated organ annotations, and metastasis number and location. Euthanasia was performed with animals at clinical terminal disease and metastatic tumor burden.

### Noninvasive imaging

A 7T Bruker Biospec (BrukerBioSpin), equipped with 35-mm inner-diameter volume coil and 12-cm inner-diameter gradients, was used for magnetic resonance imaging. A fast acquisition with relaxation enhancement sequence with 2,000/39-ms TR/TE (repetition time/echo time), 256 × 192 matrix size, r156-µM resolution, 0.75-mm slice thickness, 0.25-mm slice gap, 40 × 30-cm^2^ FOV (field-of-view), 101-kHz bandwidth and 4 NEX (number of excitation) was used for acquired in coronal and axial geometries a multi-slice T2-weighted images. All animal imaging, preparation and maintenance was carried out in accordance with MD Anderson’s Institutional Animal Care and Use Committee policies and procedures. IVIS-100 procedure has been described elsewhere^45^.

### GEKOs

#### Isolation and in vitro stabilization

Kidneys were isolated and tubular fragments were isolated by collagenase digestion (C9407, Sigma) for 30 min at 1 mg ml^−1^. Fragments were seeded in growth factor-reduced Matrigel (Corning) and cultured in medium (DMEM/F12 supplemented with 1% penicillin/streptomycin, HEPES, GlutaMAX), with 2% B27 supplement (Gibco), recombinant mouse noggin (50 ng ml^−1^, Peprotech), 10% Rspo1 (Millipore-Sigma, SCM104), EGF (50 ng ml^−1^, Peprotech), FGF-10 (100 ng ml^−1^, Peprotech), N-acetylcysteine (1.25 mM, Sigma), A8301 (5 µM, Tocris Bioscience) and primocine (0.1 mg ml^−1^, Invivogen). After 2 weeks, GEKOs were cultured using DMEM/F12 supplemented with 1% P/S (penicillin/streptomycin), 10% FBS^46^.

#### Viral transduction

Three weeks after isolation, GEKOs were dissociated from Matrigel in ice-cold PBS, collected and pelleted. Organoids were plated at high confluency in 96 wells with DMEM/F12 supplemented with 1% P/S, 10% FBS and incubated with AAV (10^7^ viral particles) or AAV + Adeno Cas9-GFP (100:1 ratio, 10^9^ viral particles and 10^7^ viral particles, respectively) for 8 h at 37 °C and 5% CO_2_. GEKOs were collected, pelleted, and embedded in Matrigel or transplanted.

### GEKTCs

#### Isolation and in vitro stabilization

Kidneys were isolated and tubular fragments were collected by collagenase digestion (C9407, Sigma) for 30 min at 0.5 mg ml^−1^ at 37 °C and 5% CO_2_. Fragments were centrifuged for 5 min at 150*g*, washed and resuspended in appropriate medium (DMEM/F12 supplemented with 1% penicillin/streptomycin, HEPES, GlutaMAX), with 1.5% B27 supplement (Gibco), recombinant mouse noggin (50 ng ml^−1^, Peprotech), 10% Rspo1 (Millipore-Sigma, SCM104), EGF (50 ng ml^−1^, Peprotech), FGF-10 (100 ng ml^−1^, Peprotech), N-acetylcysteine (1.25 mM, Sigma), A8301 (5 µM, Tocris Bioscience) and primocine (0.1 mg ml^−1^, Invivogen). After five passages, GEKTCs were cultured using DMEM/F12 supplemented with 1% P/S, 10% FBS.

#### Viral transduction

Three passages after isolation, transduction was achieved by incubating GEKTCs with AAV or AAV + Adeno Cas9-GFP for 8 h at 37 °C, 5% CO_2_ (viral concentrations as specified for GEKOs) when cells were at 50% confluency. Cell cultures were then treated with routine protocols.

### Tumor cell isolation and culture

Ex vivo cultures from primary tumor explants were generated by mechanical dissociation and incubation for 1 h at 37 °C with a solution of collagenase IV/dispase (2 mg ml^−1^) (Invitrogen), resuspended in DMEM (Lonza) and filtered. Cells derived from tumor dissociation and digestion were plated on gelatin 0.1% (Millipore-Sigma)-coated plates and cultured in DMEM (Lonza) supplemented with 20% FBS (Lonza) and 1% penicillin–streptomycin and kept in culture for five passages or less.

### Cell proliferation and clonogenic assay

Cells were seeded in a 96-well plate (500 cells per well) in medium supplemented with recombinant IFN-α 50 IU ml^−1^ or IFN-γ 50 IU ml^−1^ or vehicle. Cells were incubated at 37 °C, 5% CO_2_ in the IncuCyte (Essenbioscience) incubator. Cell confluency was measured and analyzed over a period of 5–8 d and medium was changed every 24 h.

For clonogenic assay, 100 cells were seeded in a six-well plate in medium supplemented with recombinant IFN-α 50 IU ml^−1^ or IFN-γ 50 IU ml^−1^ or vehicle and maintained at 37 °C, 5% CO_2_ (medium was changed every 24 h). After 8–15 d, clones were fixed and stained with crystal violet (0.25% crystal violet in methanol 20%). Colonies with more than approximately 50 cells were counted manually and clonogenic survival fraction was expressed as the relative plating efficiencies of the irradiated cells to the control cells.

### Protein extraction and western blot analyses

For cellular protein lysates, cells were scraped on ice using cold Ripa lysis buffer (150 nM NaCl, 50 mM Tris HCl pH 8, 1% Igepal, 0.5% sodium deoxycholate, 0.1% SDS) supplemented with a HALT protease and phosphatase inhibitor cocktail (ThermoFisher). Cell lysates were centrifuged at 17,500*g* for 20 min at 4 °C and supernatants were collected.

Proteins were separated in 4–20% SDS–PAGE (Criterion Precast Midi Gel, Bio-Rad) and transferred to nitrocellulose membranes (Trans-Blot Turbo Midi 0.2-μm nitrocellulose transfer pack, Bio-Rad). Membranes were blocked with 5% nonfat dried milk in PBS and incubated at 4 °C overnight with primary antibodies (pY701STAT1 catalog no. 9167, STAT1 catalog no. 9172, Cell Signaling Technology; H3 catalog no. sc-517576, Santa Cruz Biotechnology; tubulin catalog no. T9026, Millipore-Sigma; dilutions 1:1,000)

Membranes were washed in PBS and incubated for 1 h at room temperature with the appropriate horseradish peroxidase-conjugated secondary antibodies (Cell Signaling Technology) for ECL (enhanced chemoluminescence) detection (SuperSignal WEST Pico PLUS Chemiluminescent Substrate, ThermoFisher).

### Beta-galactosidase staining

Passage 5 GEKTCs were seeded in a six-well plate and cultured for 7 d in the presence or not of 1 µM baricitinib. Beta-galactosidase staining was performed according to the manufacturer’s protocol (Cell Signaling Technology no. 9860). Images were captured with an EVOS XL Core Imaging System.

### sgRNA design and validation

sgRNAs were designed with the GenScript CRISPR sgRNA Design Tool (https://www.genscript.com/gRNA-design-tool.html?a=post). 5′-phosphorilated oligos were annealed and diluted 1:20. Then 1 μl of each annealed and diluted sgRNA was cloned in digested lentiCRISPR V2 (Addgene no. 52961) according to Dr. Feng Zhang’s protocol (https://media.addgene.org/cms/files/Zhang_lab_LentiCRISPR_library_protocol.pdf). NEB Stable Competent *E. coli* (C3040I) colonies resistant to ampicillin antibiotic selection were amplified, and presence of sgRNA was confirmed by Sanger sequencing. Positive clones were transfected individually in 293 cells along with vectors for lentiviral packaging production, psPAX2 (Addgene no. 12260) and pMD2G (Addgene no. 12259). MCT (mouse cortical tubule) cells were infected by lentiviral particles carrying a specific sgRNA and selected for puromycin resistance. Cut efficiency of sgRNA was tested by T7 Endonuclease I (NEB no. M0302L) assay on the DNA of infected cells, according to the manufacturer’s protocol (https://www.neb.com/protocols/2014/08/11/determining-genome-targeting-efficiency-using-t7-endonuclease-i).

sgRNA sequences: *Nf2*: GTATACAATCAAGGACACGG, *Setd2*: CTCGGGTGAAAGAATATGCA, *Trp53*: GACACTCGGAGGGCTTCACT, *Cdkn2a*: GTGCGATATTTGCGTTCCGC, *Cdkn2b*: GGCGCCTCCCGAAGCGGTTC, *Bap1*: GAATCGGTCTTGCTACTGCA, *Vhl*: CGTTCCAATAATGCCCCGGA, *Ifnar1*: ACAGTTGACATAAACAAGCA, *Ifngr2*: TGGACCTCCGAAAAACATCT.

Primers list: *Nf2* For: CCTGCTTGTCTGGGAAGTCTGT, *Nf2* Rev: GTCTCACCAACTAGCCATCTTCC; *Setd2* For: TTGATTGCTGAAGGGTGTAACTCA, *Setd2* Rev: CTGGCCTCAAACTTCCTAAACAGA; *Trp53* For: CCGCCATACCTGTATCCTCC, *Trp53* Rev: GCACATAACAGACTTGGCTG; *Cdkn2a* For: AAGGGCAGGGTGTAGAGTAAC, *Cdkn2a* Rev: CAGGTGATGATGATGGGCAA; *Cdkn2b* For: GGAATTAAGTGCTGGGTTGGAG, *Cdkn2b* Rev: CAGGACGCTCACCGAAGCTA; *Bap1* For: GCCAGAACCACGTCACCTTC, *Bap1* Rev: CAGGCCACAGGCAACCTAAA.

### Recombinant DNA

Packages of two or more guide RNAs were designed and synthetized according to the following scheme: EcorI restriction site – U6 promoter – gRNA1 sequence – gRNA scaffold – polyA – U6 promoter – gRNAn sequence – gRNA scaffold – polyA – AscI restriction site. The synthetic sequence was assembled into the pEMS2158-FLEx-Flpo AAV vector (Genscript) into the EcorI and AscI restriction sites. The pEMS2158-FLEx-Flpo was generated by PCR amplification of FLEx(loxP)-FlpO from the pTCAV-FLEx(loxP)-FlpO vector (Addgene no. 67829) and cloned into the AscI and BsrGI sites of the pEMS2158 vector (Addgene no. 70119). AAV PHP.eB (Addgene no. 28304-PHPeB) carrying FLEX-GFP sequence was used for injections in Pax8^Cre/+-^Rosa26^LSL-FSF-TdT/LSL-Luc^ mice.

### Virus production

Plasmid DNA preparations were generated using endotoxin-free MIDI kits (Qiagen). Large-scale AAV particle production was outsourced to Vigene Biosciences (10^13^ IU ml^−1^). Viral preparations were stored in aliquots at −80 °C. Lentiviral particles were produced using psPAX2 and pMD2G helper plasmids. For transfection, 293T cells were cultured in DMEM containing 10% FBS (Gibco), 100 IU ml^−1^ penicillin (Gibco), 100 μg ml^−1^ streptomycin (Gibco) and 4 mM caffeine (Sigma Aldrich) and transfected using the polyethyleneimine method. Virus-containing supernatant was collected 48–72 h after transfection, spun at 3,000 r.p.m. for 10 min and filtered through 0.45-μm low-protein-binding filters (Corning). High-titer preparations were obtained by multiple rounds of ultracentrifugation at 23,000 r.p.m. for 2 h each. Adeno Cas9-GFP was purchased from Vector Biolabs (catalog no. 1901).

### Staining

Immunohistochemistry (IHC) and immunofluorescence were performed as previously described^45^. Antibodies list: RFP (ThermoFisher, catalog no. MA5-15257, 1:100 dilution), GFP (Abcam, catalog no. 13970, 1:100 dilution), Vimentin (Abcam, catalog no. ab8978, 1:200 dilution), Pax8 (Proteintech, catalog no. 10336-1-AP, 1:200 dilution), CD31 (Cell Signaling, catalog no. 77699S, 1:100 dilution), Ki67 (ThermoFisher, catalog no. MA5-14520, 1:500 dilution), cGAS (Cell Signaling, catalog no. 31659S, 1:50 dilution).

Multispectral imaging using the Vectra Microwave treatment was applied to perform antigen retrieval, quench endogenous peroxidases and remove antibodies from earlier staining procedures. The slides were stained with primary antibodies against RFP, Pax8 and Vimentin, and TSA (tyramide signal amplification) dyes to generate Opal signal (vimentin, Opal 570; RFP, Opal 620; and Pax8, Opal 690). The slides were scanned with the Vectra 3 image scanning system (Caliper Life Sciences), and signals were unmixed and reconstructed into a composite image with Vectra inForm software 2.4.8.

GEKOs were disaggregated using Trypsin to obtain a single-cell suspension and 10,000 GEKO-derived cells were embedded in 10% Phenol Red Free Reduced Growth Factor (GFR) Matrigel (Corning) mixed with the GEKO medium and layered on top of a bottom layer of polymerized GFR–Matrigel, in an eight-well Labtek chamber slide (Becton Dickinson). Embedded cells were incubated at 37 °C for 1 week. When the GEKOs were fully formed, morphological assessments were carried out using immunofluorescent staining. GEKOs were fixed in 4% PFA for 30 min at room temperature, permeabilized using 1 × PBS and 0.1%Triton X-100 for 10 min at room temperature, and washed twice with 1 × PBS for 30 min at room temperature, and then blocked using 1 × PBS, 5% goat serum, 0.1%Triton X-100, 3% BSA. Primary antibodies (Pax8 and GFP) were incubated ON at 4 °C. Secondary antibodies (AlexaFluor 488-, 594-conjugated, Invitrogen) were incubated for 1 h at room temperature, and nuclei were counterstained using DAPI for 10 min at room temperature. Samples were analyzed using a confocal laser-scanning microscope (TSP8, Leica) interfaced with a Leica fluorescent microscope. Collected images were analyzed using the LAS (Leica) software.

Estimation of purity was calculated as percentage of positive area for TdTomato (TdT) IHC staining. IHC Profiler was used for quantification of TdT% (ref. ^47^).

### Metaphase spread and chromosome count

Immunofluorescence on metaphasic spread was obtained as previously described with few modifications^48^. Cultures were treated with 100 ng ml^−1^ nocodazole for 8 h overnight, collected by trypsinization, resuspended in 0.2% (w/v) KCl and 0.2% (w/v) trisodium citrate hypotonic buffer at room temperature (20–22 °C) for 10 min and cytocentrifuged onto SuperFrost Plus glass slides (MenzelGlaser) at 450*g* for 10 min in a Shandon Cytospin 4. Slides were fixed at room temperature for 10 min in 1 × PBS with 4% (v/v) formaldehyde, permeabilized for 10 min at room temperature in KCM buffer (120 mM KCl, 20 mM NaCl, 10 mM Tris (pH 7.5) and 0.1% (v/v) Triton X-100) and blocked with 5% goat serum, 1 × PBS, 0.1% Triton and 100 × BSA 3% for 30 min at room temperature. Slides were incubated with primary antibody diluted in antibody dilution buffer (1 × PBS 0.1% Triton, 100 × BSA 3%) for 1 h at room temperature, washed in 1 × PBST (1 × PBS with 0.1% (v/v)), incubated with secondary antibody diluted in antibody dilution buffer for 30 min at room temperature, washed with 1 × PBST and stained for DNA with DAPI. Primary antibody: anti-centromere (1:250; Antibodies Incorporated). Secondary antibody: goat anti-human conjugated to AlexaFluor 488 (1:500; A-11013).

### NGS of murine DNA

Exome libraries and whole-genome libraries were prepared using a modified protocol^49^. Modifications included: the use of 1,000 ng of treated gDNA, performing only six cycles of PCR amplification and usage of the Agilent SureSelectXT Mouse All Exon Kit for exon target capture. For murine WGS, after adapter ligation, libraries were only amplified by two cycles of PCR. Equimolar quantities of the whole-genome indexed libraries were multiplexed, with 18 libraries per pool. Results from 13 of the 18 libraries were used in our analysis. All pooled libraries were sequenced on an Illumina NovaSeq6000 using the 150-base pair (bp) paired-end format.

### Bioinformatic processing of high-throughput sequencing data

The bioinformatic processing pipeline of raw WES and WGS high-throughput sequencing data was adapted for murine data from Seth et al.^50^. Reads were aligned to the mouse genome reference (mm10) using Burrows–Wheeler Aligner with a seed length of 40 and a maximum edit distance of 3 (allowing for distance % 2 in the seed)^51^. BAM files were further processed according to GATK Best Practices, including removal of duplicate reads, realignment around indels and base recalibration^52^.

### Analysis of sgRNA performanc*e*

Expected cut sites of sgRNAs were analyzed using CRISPResso2 (ref. ^53^). BAM files were first filtered with SAMtools^51^ to contain reads spanning a 50-bp region centered around the expected sgRNA cut site and passed to CRISPResso2 in ‘CRISPRessoWGS’ mode. The allele frequency of each base position around the cut site window was extracted from the CRISPResso2 results. An odds ratio for probability of a base position difference from the reference genome for each tumor sample and its respective matched normal sample was calculated by Fisher’s exact test by counting the number of base alterations observed at each cut site window position. The odds ratios were transformed by natural log and z-transformation against the average log-odds ratio for all base positions of the same gene. The z-transformed log-odds ratios were then averaged across all gene cut sites for a sample to summarize the overall editing efficiency of the sgRNAs delivered to each mouse^30^. Genes were considered altered if at least two reads with the same pattern of base alteration were detected at the expected sgRNA cut site and if coverage of the envisaged targeted region was inferior to 50% of the region median coverage of a healthy control.

### Identification and characterization of somatic mutations

Somatic mutations were detected from murine tumor samples using a combination of MuTect v.1 (ref. ^54^) to call somatic SNVs and Pindel^55^ to call somatic insertions and deletions (indels). Tumor samples from both WES and WGS were compared with their respective matched control. All mutations were also filtered for depth (tumor sample coverage >20×, normal sample coverage >10×) and VAF (VAF ≥ 0.1). Additional filters for Pindel calls were implemented due to a known false-positive bias for Pindel. VAFs were also re-derived for Pindel calls across all samples by interrogating reads from BAM files. The 20 nucleotides immediately following each Pindel call were also examined to confirm that no nucleotide sequence (length ≤ 6) was repeated more than two times, eliminating false-positive indel calls that may happen in highly repetitive regions. All mutations annotated to genomic regions not targeted by an sgRNA detected in at least one sample were kept.

Mutation patterns of WGS samples were then determined by extracting all passing somatic SNVs as called by Mutect v.1 and mapped to the corresponding pyrimidine trinucleotide context-specific somatic SNV. Duplicate mutations in different samples originating from the same mouse were removed, and then the frequency of each trinucleotide context-specific mutation for each mouse sample cohort of metastatic samples or of primary tumors plus cell line samples was calculated. Comparative analyses of mutational signatures in human and murine tumors were performed according to Alexandrov et al.^15^. The counts across all trinucleotide context-specific somatic SNVs were then summed across the entire cohort and frequencies calculated for the entire summed cohort.

### Identification of somatic copy number profiles and events

CNVkit^56^ was used to derive somatic copy number profiles from WES data using a panel of normal samples consisting of all the matched normal samples across all mice sequenced in this study. The targeted exome bed file for the Agilent SureSelect All Mouse Exon V1 was downloaded from Agilent with the original mm9 coordinates and was then converted to mm10 using CrossMap v.0.3.4 for use by CNVkit. Occurrences of CNVs in focal regions of the genome were called if all exons spanning the region of interest had an absolute weighted average log_2_ read-depth ratio of ≥0.4. Otherwise, GISTIC2 was run with amplification and deletion thresholds of 0.2, using gene-level assumptions for significance, along with additional broad-level analysis. The GISTIC2 reference genome file for mm10 was acquired, and no marker file was necessary^57,58^.

Sequenza^59^ was used to derive somatic copy number profiles from WGS data using each sample’s matched normal sample. To assign ploidy to WGS samples, purity was first estimated by TdT protein, and the ploidy with the largest predicted probability at the estimated purity was selected from the Sequenza cellularity–ploidy prediction table.

### Construction of tumor progression sample tree representation

The sample progression tree representation of tumors was constructed with hierarchical clustering using the complete linkage algorithm and the hamming distance between samples. The hamming distance was calculated as the number of nondriver somatic mutations shared by any two samples as a fraction of the total number of nonsomatic mutations contained by either sample. Visualizations of sample progression trees were manually generated. Branch lengths of 0 were collapsed to the direct ancestor node. Only mutations detected in all descendants of a branch were considered.

### Statistical analysis of clinical RCC cohort data

Processed clinical, copy number, somatic mutation and molecular characterization data from the TCGA pan-kidney (TGCA-KIPAN) tumor sample cohort were obtained from Ricketts et al.^11^. TCGA profiling data were then augmented with arm-level copy number calls, aneuploidy score and WGD status as determined by Taylor et al.^56^. The aneuploidy score was then transformed to calculate a fraction of genome altered (fCNA) as described by Taylor et al.^60^. TCGA tumors with sarcomatoid features were manually annotated as described by Bokouny et al.^21^. Clinical data used for confirmation of genomic effects of 9p loss on WGD and aneuploidy were acquired from the TRACERx renal cell cancer cohort and an RCC cohort from the Memorial Sloan Kettering Cancer Center kidney cancer cohort (MSK-Met)^3,11^.The aneuploidy score for TRACERx samples was calculated using the arm-level chromosome alteration calls from TRACERx directly and then converted to an fCNA value as described by Taylor et al^60^.

### B-allele frequency comparison

Murine B-allele frequencies (BAFs) were calculated using the snp-pileup script from the FACETS software package on WGS samples^61^. The VCF of identified murine SNP locations was obtained from the Wellcome Sanger Institute, Mouse Genome Project v.5, dbSNP142 (ref. ^62^). The snp-pileup counts were then utilized to determine the allele frequencies of these common murine SNPs. Heterozygous SNPs were identified if the BAF (alternative nucleotide) was 0.2 < BAF < 0.8, with minimum coverage of 15× in the normal tumor sample. BAFs of heterozygous SNPs identified in each mouse’s normal tissue sample were plotted against corresponding tissue sample BAFs for the same SNP.

### Single-cell sequencing sample and library preparation

GEKOs were dissociated from Matrigel and resuspended as single-cell suspensions in 1 × PBS, 2.5% FBS solution for further processing. Chromium single-cell sequencing technology from 10x Genomics was used to perform single-cell separation, complementary DNA amplification and library construction. Cellular suspensions were loaded on a 10x Chromium Single Cell Controller to generate single-cell gel bead-in-emulsions. The scRNA-seq libraries were constructed using the Chromium Single Cell 3ʹ Library & Gel Bead Kit v.2 (PN-120237, 10x Genomics). The HS dsDNA Qubit Kit was used to determine the concentrations of both the cDNA and the libraries. The HS DNA Bioanalyzer was used for quality-tracking purposes and size determination for cDNA and lower-concentrated libraries. Sample libraries were normalized to 7.5 nM and equal volumes were added of each library for pooling. The concentration of the library pool was determined using the Library Quantification qPCR Kit (KAPA Biosystems) before sequencing. The barcoded library at the concentration of 275 pM was sequenced on the NovaSeq6000 (Illumina) S2 flow cell (100 cycle kit) using a 26 × 91 run format with 8-bp index (read 1). To minimize batch effects, the libraries were constructed using the same versions of reagent kits and following the same protocols, and the libraries were sequenced on the same NovaSeq6000 flow cell and analyzed together.

### scRNA-seq data processing and analysis

The raw scRNA-seq data were preprocessed (demultiplex cellular barcodes, read alignment and generation of gene count matrix) using the Cell Ranger Single Cell Software Suite. Genes detected in fewer than three cells and cells with low-complexity libraries (in which detected transcripts were aligned to fewer than 350 genes) were filtered out and excluded from subsequent analysis. Low-quality cells with >25% of mitocondrial transcripts were considered apoptotic and excluded. Following removal of the poor-quality cells, a total of 87,718 cells were retained for downstream analyses. Library size normalization was performed in Seurat^19^ on the filtered gene–cell matrix to obtain the normalized UMI (unique molecular identifier) count data. Cluster analysis, group determination and cluster distribution among different experimental groups were performed with the Seurat package^19^. The cell-cycle stage was computationally assigned for each individual cell by the Seurat function CellCycleScoring. Cell-cycle signature, EMT (epithelial-to-mesenchymal transition) signature and interferon score were calculated based on the expression profiles of three publicly available signatures (‘KEGG_CELL_CYCLE’, ‘HALLMARK_EPITHELIAL_MESENCHYMAL_TRANSITION’, ‘HALLMARK_INTERFERON_ALPHA_RESPONSE’). Monocle 3 alpha^20^ was applied as an independent tool for unsupervised trajectory analysis and three-dimensional graphs were generated using Monocle 3 reduce dimension and plot dimensions for dimensionality reduction and visualization. Pseudotime was calculated with Monocle 3 functions. Inferred CNVs from scRNA-seq data were generated with inferCNV (https://github.com/broadinstitute/inferCNV) and the following parameters: wild-type organoids were used as a normal reference; cutoff was set at 0.1; minimum cells per gene equal to 3.

### Genome-wide CRISPR screening

Briefly, lentiviral particles of the mouse genome-wide CRISPR library (mTKOv3) were generated by the University of Michigan Biomedical Research Lentiviral Core and concentrated 100×. Cells were transduced with the mouse genome-wide CRISPR library in 500-cm^2^ square dishes (Corning) with 8 μg ml^−1^ polybrene (Santa Cruz Biotechnology) at MOI (multiplicity of infection) of 0.3 and an estimated 400× coverage. The medium was replaced 24 h after infection and after another 24 h puromycin selections were started. After 72 h, cells were trypsinized, pooled together and counted. As a reference, 30 × 10^6^ cells were immediately collected. Every passage of 15 × 10^6^ cells (~200× coverage) was maintained in culture until the endpoint (20 doublings) when 30 × 10^6^ cells (~400× coverage) were collected.

The cell pellets were suspended in 2 ml of Buffer P1/RNAse A and lysed by adding 1/20 volume of 10% SDS (Promega). After mixing and 10 min of incubation at room temperature, the genomic DNA (gDNA) was sheared by passing the lysate 10–15 times through a 22-gauge syringe needle. Then, the first extraction step was executed by adding 1 volume of phenol:chloroform:isoamyl alcohol (25:24:1, molecular biology grade (Sigma Aldrich)) to the lysate. The samples were centrifugated at 17,000*g* for 10 min and the upper phase was moved to a new tube. Then, the second extraction step with chloroform:isoamyl alcohol (24:1 (Sigma Aldrich)) was performed. Afterwards, the upper phase was transferred to a new tube and mixed with 0.1 volumes of 3 M NaCl (Sigma Aldrich) and 0.8 volumes of 2-propanol (Fisher Scientific) to precipitate the gDNA. The samples were centrifugated at 17,000*g* for 20 min at 4 °C and then the DNA pellet was washed once in 70% ethanol (Fisher Scientific) and centrifuged again for 5 min at 17,000*g* at 4 °C. The DNA pellet was then dried and resuspended overnight in UltraPure distilled water (Invitrogen). The gDNAs were quantified by NanoDrop 2000 (Thermo Scientific). For the generation of NGS libraries, barcodes were amplified in two rounds of PCR using the Titanium Taq DNA polymerase (Clontech-Takara). The first PCR reactions contained 10 µg of gDNA per PCR reaction and the total reactions resulted in targeted amplification from a third of the total gDNA. The first 16 cycles targeted PCR amplification and utilized the following primer set: mTKOv3-PCR1-F: ATTAGTACAAAATACGTGACGTAGAA and mTKOv3-PCR1-R: ACCTTCTCTAGGCACCGGATCA. The second PCR reactions were performed for 14 cycles using the following primers with adapters optimized to introduce the specific adapters for Illumina NGS technology specific for the Hiseq4000: mTKO-P2-F: AATGATACGGCGACCACCGAGATCTACACGAGATCGGACTATCATATGCTTACCGTAACTTGAA and mTKO-P7##-IND: CAAGCAGAAGACGGCATACGAGATGCACGACGAGACGCAGACGAAnnnnnAGAGCAACTTCTCGGGGACTGTGGGCGA. Amplified PCR products from two replicates of the second PCR reactions were pooled together and extracted from agarose gel with the QIAquick gel purification kit (QIAGEN). Samples were quantified using Qubit 2.0 DNA HS Assay (ThermoFisher), QuantStudio 5 System (Applied Biosystems) and Tapestation High Sensitivity D1000 Assay (Agilent Technologies). Six samples were pooled equilmolar to be run on a Nextseq 500 high-output 75-bp SR with 10% PhiX. Custom primers were required for Read 1 (20 nt): mTKO-Seq-26bp TCTTGGCTTTATATATCTTGTGGAAAGGACGAAACACCG, and to obtain the sample index, Read 2 (6 nt): mTKO-Seq-Index-7 AGATGCACGACGAGACGCAGACGAA.

#### Bioinformatic analysis

Bowtie^63^ was used to obtain raw read-counts for each screen, with 1 mismatch allowance, taking the best-matching sgRNA per read. Following this, BAGEL2 (ref. ^64^) software was used to calculate normalized read-counts, and log_2_ foldchange was obtained for each screen compared with the reference timepoint of the corresponding cell line. Next, TSGs were determined by identifying the genes with the highest log_2_ foldchange in each cell line model. The top 2,000 log_2_ foldchange ranked TSGs were used as an input for Enrichment Pathway Analysis using Reactome and Panther databases.

### Summary of methods for RCC MSK cohort

RCC tumor specimens from 134 patients were procured from the Memorial Sloan Kettering (MSK) Pathology Department after ethics review board approval. Primary and metastatic deposit specimens were reviewed by a specialized genitourinary pathologist. Clinicopathologic and molecular data for 62 of these patients have been reported in a previous publication^6^.

Macro-dissected tumor and paired adjacent normal kidney tissue or blood were sent for DNA extraction and sequencing at the Integrated Genomic Operations Core of MSK or the Molecular Diagnostics Service laboratory of the Department of Pathology. Sequencing was done on both the tumor and matched normal samples using the MSK-IMPACT gene panel (MSK-IMPACT)^65^. Samples were sequenced at an average depth of 500×.

Raw sequencing data were aligned to a reference genome (b37) and somatic variants were called using a previously validated pipeline. Briefly, four different variant calling tools were used for this purpose: MuTect2 (part of GATK v.4.1.4.1)^52^, Strelka2 v.2.9.10 (ref. ^66^), Varscan v.2.4.3 (ref. ^67^) and Platypus^68^. Ancillary filters were then applied to obtain high-accuracy mutations; these included: a coverage of at least 10 reads in the tumor, with 5 or more supporting the variant of interest, a VAF ≥ 5% in the tumor and a VAF < 7% in the matched normal sample. Only somatic nonsynonymous exonic mutations were considered, and SNVs identified at a frequency >1% in dbSNP or 1000Genomes projects were removed. All variant calls were manually reviewed by investigators for additional accuracy.

Allele-specific copy number analysis and purity estimation were done using the FACETS algorithm v.0.5.6. Inference of arm-level and genome-doubling events was performed using a public R package (https://github.com/mskcc/facets-suite). All CNVs in autosomal chromosomes were considered, regardless of length. Informed consent was obtained after the nature and possible consequences of the studies were explained.

### Analysis of the CCLE

Data were retrieved from the DEPMAP database (https://depmap.org/portal/). Tumor cell lines from solid tumors were included in the analysis and divided into ‘low’ (lower quartile) and ‘high’ (upper quartile) aneuploidy score and compared for log copy number values.

### Statistics and reproducibility

Data are presented as the mean or median ± s.d. and percentages. Comparisons among biological replicates were performed using two-tailed Student’s *t*-test, two-way analysis of variance (ANOVA) and Mann–Whitney *U* test. Results from survival experiments were analyzed with log-rank (Mantel–Cox) test and expressed as Kaplan–Meier survival curves. Results from contingency tables were analyzed using two-tailed Fisher’s exact test or chi-squared test for multiple comparisons. All of the statistical analyses were performed with GraphPad Prism software. Data distribution was assumed to be normal without formal testing. Group size was determined on the basis of the results of preliminary experiments. No statistical methods were used to determine sample size. No data were excluded from the analysis. Group allocation and analysis of outcome were not performed in a blinded manner, with the exception of in vivo treatment with baricitinib. In vitro experiments were repeated three times, while in vivo experiments were performed at least twice.

### Reporting summary

Further information on research design is available in the Nature Portfolio Reporting Summary linked to this article.

## Supplementary information

Supplementary Information Raw western blot figures.

Reporting Summary

Supplementary Table 1 Clinical data for MSKCC TRACERX and TCGA cohorts and WES and clinical information of SM-GEMM.

## Data Availability

All data supporting the findings of this study are available within the article and its Supplementary Information. Murine genomic and single-cell RNA-seq raw data have been deposited in the Sequence Read Archive (SRA) under accession code: PRJNA835458. Previously published datasets and information info are available with the following links and accession codes: 10.6084/m9.figshare.21637199.v2 (Broad DepMap (2022): DepMap 22Q4 Public); EGAS00001002793 (TRACERx genomic data)^3^; http://cancergenome.nih.gov/ (TCGA Research Network, pan-kidney transcriptomic, genomic and clinical data); GSE85971 (MSKCC genomic data). Requests for resources and reagents can be directed to the lead contact G.G. Source data are provided with this paper.

## Source data

Source Data Fig. 1 Statistical source data.
Source Data Fig. 2 Statistical source data.
Source Data Fig. 3 Statistical source data.
Source Data Fig. 4 Statistical source data.
Source Data Fig. 5 Statistical source data.
Source Data Fig. 6 Statistical source data.
Source Data Extended Data Fig. 2 Statistical source data.
Source Data Extended Data Fig. 4 Statistical source data.
Source Data Extended Data Fig. 5 Statistical source data.
Source Data Extended Data Fig. 6 Statistical source data.
Source Data Extended Data Fig. 7 Statistical source data.
Source Data Extended Data Fig. 8 Statistical source data.
Source Data Extended Data Fig. 9 Statistical source data.
Source Data Extended Data Fig. 10 Statistical source data.

## Code availability

Codes used for this manuscript have been previously published and adequately referenced in this manuscript. Methodological details on parameters used are available in the Methods section of this manuscript.
