# Supplementary material for: Interferon signaling promotes tolerance to chromosomal instability during metastatic evolution in renal cancer
Source: Nat Cancer. 2023 Jun 26;4(7):984–1000. doi: 10.1038/s43018-023-00584-1 (PMC10368532; doi:10.1038/s43018-023-00584-1)

nature cancer

Article https://doi.org/10.1038/s43018-023-00584-1

Interferon signaling promotes tolerance to chromosomal instability during metastatic evolution in renal cancer

In the format provided by the authors and unedited

Raw data for Supplementary Figure 11

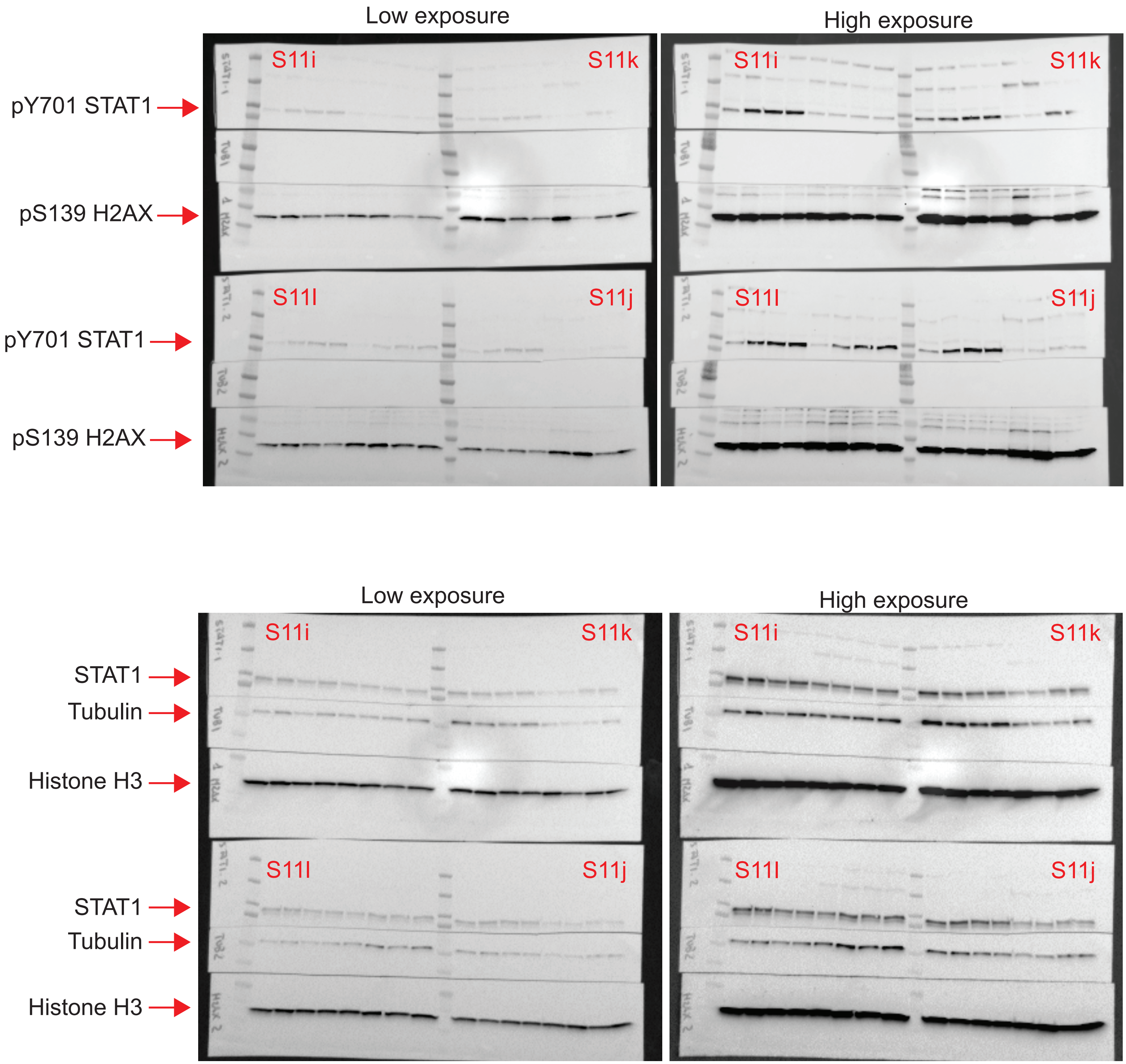

Supplement: Supplementary file 1 — Raw western blot figures. [file 43018_2023_584_MOESM1_ESM.pdf]
