# Supplementary material for: Interferon signaling promotes tolerance to chromosomal instability during metastatic evolution in renal cancer
Source: Nat Cancer. 2023 Jun 26;4(7):984–1000. doi: 10.1038/s43018-023-00584-1 (PMC10368532; doi:10.1038/s43018-023-00584-1)
Supplement: Supplementary file 2 — Reporting Summary [file 43018_2023_584_MOESM2_ESM.pdf]

nature portfolio

Corresponding author(s): Luigi Perelli, Alessandro Carugo, Giannicola Genovese

Last updated by author(s): 2023/05/17

Reporting Summary

Nature Portfolio wishes to improve the reproducibility of the work that we publish. This form provides structure for consistency and transparency in reporting. For further information on Nature Portfolio policies, see our Editorial Policies and the Editorial Policy Checklist.

Statistics

For all statistical analyses, confirm that the following items are present in the figure legend, table legend, main text, or Methods section.

n/a	Confirmed	
☐	☒	The exact sample size (*n*) for each experimental group/condition, given as a discrete number and unit of measurement
☐	☒	A statement on whether measurements were taken from distinct samples or whether the same sample was measured repeatedly
☐	☒	The statistical test(s) used AND whether they are one- or two-sided<br>*Only common tests should be described solely by name; describe more complex techniques in the Methods section.*
☐	☒	A description of all covariates tested
☐	☒	A description of any assumptions or corrections, such as tests of normality and adjustment for multiple comparisons
☐	☒	A full description of the statistical parameters including central tendency (e.g. means) or other basic estimates (e.g. regression coefficient) AND variation (e.g. standard deviation) or associated estimates of uncertainty (e.g. confidence intervals)
☐	☒	For null hypothesis testing, the test statistic (e.g. *F*, *t*, *r*) with confidence intervals, effect sizes, degrees of freedom and *P* value noted<br>*Give P values as exact values whenever suitable.*
☒	☐	For Bayesian analysis, information on the choice of priors and Markov chain Monte Carlo settings
☒	☐	For hierarchical and complex designs, identification of the appropriate level for tests and full reporting of outcomes
☒	☐	Estimates of effect sizes (e.g. Cohen's *d*, Pearson's *r*), indicating how they were calculated

Our web collection on statistics for biologists contains articles on many of the points above.

Software and code

Policy information about availability of computer code

Data collection	Software and code reported in this work for data colletion are available online and published elsewhere. For each code used in this work, we included its reference.
Data analysis	Software and code reported in this work for data analysis are available on line and published elsewhere. For each code used in this work, we included its reference. Softwares for data analysis: Vectra inForm software 2.4.8. LAS X (Leica) v1.4.4.

For manuscripts utilizing custom algorithms or software that are central to the research but not yet described in published literature, software must be made available to editors and reviewers. We strongly encourage code deposition in a community repository (e.g. GitHub). See the Nature Portfolio guidelines for submitting code & software for further information.

Data

Policy information about availability of data

All manuscripts must include a data availability statement. This statement should provide the following information, where applicable:

- Accession codes, unique identifiers, or web links for publicly available datasets
- A description of any restrictions on data availability
- For clinical datasets or third party data, please ensure that the statement adheres to our policy

All data supporting the findings of this study are available within the Article and its Supplementary Information. Murine genomic and single cell RNA seq raw data

have been deposited in the Sequence Read Archive (SRA) under accession code: PRJNA835458. Source data are provided with this paper. Requests for resources and reagent can be directed to the lead contact G.G.

Research involving human participants, their data, or biological material

Policy information about studies with human participants or human data. See also policy information about sex, gender (identity/presentation), and sexual orientation and race, ethnicity and racism.

Reporting on sex and gender	No human participants have been enrolled in this study. The findings of this study, extrapolated and tested on available databases cited accordingly, have no sex or gender biasese.
Reporting on race, ethnicity, or other socially relevant groupings	N/A
Population characteristics	N/A
Recruitment	N/A
Ethics oversight	N/A

Note that full information on the approval of the study protocol must also be provided in the manuscript.

Field-specific reporting

Please select the one below that is the best fit for your research. If you are not sure, read the appropriate sections before making your selection.

☒ Life sciences ☐ Behavioural & social sciences ☐ Ecological, evolutionary & environmental sciences

For a reference copy of the document with all sections, see nature.com/documents/nr-reporting-summary-flat.pdf

Life sciences study design

All studies must disclose on these points even when the disclosure is negative.

Sample size	Sample size for each experiment is reported in the Article and attached supplementary information. No statistical methods were used to calculate and predetermine sample size. For in vitro experiments, we used a minimum sample size with N = 3; for in vivo experiments, we included a minimum of N=5 mice per each experimental group. Sample size was determined on the basis of previous experiments.
Data exclusions	No data were excluded from this work.
Replication	In vitro experiments were repeated twice and all attempts were successful. In vivo experiments were performed at least twice. All the attempts of replication were successful for in vitro studies.
Randomization	Group allocation was randomized in regards of sex for in vivo experiment. Group allocation was not randomized in regards of genotype and age; specifically 4-6 weeks old mice were included in all the in vivo studies. Specific genotypes were included for in vivo experiments.
Blinding	Group allocation during data collection was not performed in a blinded manner, with the exception of Baricitinib treatment in vivo experiments. Briefly, investigator LP transplanted 16q-euploid and 16q-loss SM-GEMM RCC derived cell lines and Nf2-Setd2-4q Ts65Dn and Wild Type GEKOs in recipient immunocompromised mice; investigator CL was blinded for the genotypes of these groups and performed Baricitinib treatment. Analysis of outcomes was not performed in a blinded manner. Blinding was not performed as for the difficulty to achieve reproducibility of animal experiments in terms of genotypes and age.

Reporting for specific materials, systems and methods

We require information from authors about some types of materials, experimental systems and methods used in many studies. Here, indicate whether each material, system or method listed is relevant to your study. If you are not sure if a list item applies to your research, read the appropriate section before selecting a response.

Materials & experimental systems

n/a	Involved in the study
☐	☒ Antibodies
☐	☒ Eukaryotic cell lines
☒	☐ Palaeontology and archaeology
☐	☒ Animals and other organisms
☒	☐ Clinical data
☒	☐ Dual use research of concern
☒	☐ Plants

Methods

n/a	Involved in the study
☒	☐ ChIP-seq
☒	☐ Flow cytometry
☒	☐ MRI-based neuroimaging

Antibodies

Antibodies used

RFP (Thermo Fisher, cat. #MAS-15257, 1:100 dilution), GFP (Abeam, cat. #13970, 1:100 dilution), Vimentin (Abeam, cat. #ab8978, 1:200 dilution), Pax8 (Proteintech, cat.
#10336-1-AP, 1:200 dilution), CD31 (Cell Signaling, cat.# 77699S, 1:100 dilution), Ki67 (Thermo Fisher, cat. #MAS-14520, 1:500 dilution) cGAS (Cell Signaling, cat.# 31659S, 1:50 dilution), pY701STAT1 (cat. #9167 Cell Signaling Technology, 1:1000 dilution), STATl (cat. #9172, Cell Signaling Technology, 1:1000 dilution), H3 (cat. sc-517576, Santa Cruz Biotechnology, 1:1000 dilution); tubulin (cat #T9026, Millipore-Sigma, 1:1000 dilution). AlexaFluor 488 Goat anti-Human IgG (dilution: 1:500 - cat. # A-11013, ThermoFisher). Anti-centromere (SKU: 15-234 AntibodiesInc, dilution 1:250).

Validation

RFP MAS-15257 has successfully been used in immunoprecipitation, ELISA, Western blotting, immunohistochemistry, im munocytochem istry and i mm unofluorescence applications (https://www.thermofisher.com/a ntibody /product/RF P-Anti body-clone-RFSR-Monoclonal/MAS-15257); GFP 13970 has successfully been used for Western Blotting and immunofluorescence (https://www.abcam.com/gfp-antibody-ab13970.html). Vimentin ab8978 has been successfully used for Western Blotting, immunohistochemistry, immunofluorescence and Flow Cytometry (https://www.abcam.com/vimentin-antibody-rv202-cytoskeleton-marker-ab8978.html); Pax8 10336-1-AP has been successfully used for Western Blotting, lmmunohistochemistry and lmmunofluoresce (https://www.ptglab.com/products/PAX8-Antibody-10336-1-AP.htm#tested-applications). CD31 77699S has been successfully used for Western Blotting, lmmunohistochemistry, lmmunofluorescence (https://www.cellsignal.com/products/ primary-antibodies/cd31-pecam-1-d8v9e-xp-rabbit-mab/77699); Ki67 MAS-14520 has successfully used for Western Blotting, immunohistochemistry, lmmunofluorescence, Flow Cytometry (https://www.thermofisher.com/antibody/product/Ki-67-Antibody-clone-SP6-Recombinant-Monoclonal/MA5-14520); cGAS 31659S has been successfully used for Western Blotting and Immunoprecipitation (https ://www. ce I I sign a I. com/ products/ primary-anti bodies/ cgas-d 3080-ra b bit-ma b-mo use-speci fic/31659). pY701STAT1 9167 has bee successfully used for Western Blotting, lmmunoprecipitation, lmmunohistochemistry, Flow Cytometry
(https://www.cellsignal.com/products/primary-antibodies/phospho-statl-tyr701-58d6-rabbit-mab/9167); STA Tl 9172 has been successfully used for Western Blotting, lmmunoprecipitation, lmmunohistochemistry, Flow Cytometry (https://www.cellsignal.com/ products/primary-antibodies/statl-antibody/9172); H3 sc-517576 has been successfully used for Western Blotting (https:// www.scbt.com/p/histone-h3-antibody-1g1); tubulin T9026 has been successfully used for Western Blotting (https:// www.sigmaaldrich.com/US/en/product/sigma/t9026).

Eukaryotic cell lines

Policy information about cell lines and Sex and Gender in Research

Cell line source(s)

293T cell line was purchased by ATCC (CRL-3216). All other cell lines used in this work where generated in our lab from SM-GEMM RCC model and they are available upon reasonable request by contacting the leasing author GG. 16q loss cell line was derived from a male mouse. 16q euploid cell line was derived from a female mouse.

Authentication

Cell lines were not authenticated from a third party, with the exception of the 293T (authentication with morphological observation). Cell lines are available upon reasonable request by contacting the leading author GG.

Mycoplasma contamination

Cells were tested for mycoplasma contamination at least once via PCR with negative results.

Commonly misidentified lines
(See ICLAC register)

No commonly misidentified cell lines were used in this study

Animals and other research organisms

Policy information about studies involving animals; ARRIVE guidelines recommended for reporting animal research, and Sex and Gender in Research

Laboratory animals

The Pax8Cre strain was generated by Dr. Mein rad Busslinger and obtained through the Jackson Laboratory, Stock No: 028196. The H11LSL-Cas9 strain was generated by Dr. Monte M. Winslow and obtained through the Jackson Laboratory, Stock No: 027632. The Rosa26LSL-TdT was generated in Dr. Hongkui Zeng's laboratory and obtained through the Jackson Laboratory, Stock No: 007908. The Rosa26fsf-lsl-TdTomato was generated in Hongkui Zeng's lab and obtained through the Jackson Laboratory, Stock No: 021875. Rosa26LSL-Luc mice were generated by Dr. William G. Kaelin and obtained through the Jackson Laboratory, Stock No: 034320. The Ts65Dn strain was generated by Dr. Muriel T Davisson and obtained through the Jackson Laboratory, Stock No: 001924. Strains were kept in a mixed C57BL/6 and 129Sv/Jae background, except for the Ts65Dn that was kept in B6EiC3Sn background. Embryo collection

was performed at El 4. CBl 7SC-F SCID mice were purchased from Taconic. All mice allocated into experimental groups were between 4-6 weeks of age. Mice were kept at a 12 light/12 dark cycle is commonly used, housed at 18-23 degree Celsius and humidity of 50-60%. Maximal tumor burden was not exceeded according to the institutional review board guidelines.

Wild animals	No wild animals were included in this study.
Reporting on sex	No sex specific findings were reported.
Field-collected samples	No field-collected samples were used in this study.
Ethics oversight	All animal studies and procedures were approved by the UTMDACC Institutional Animal Care and Use Committee. All experiments conformed to the relevant regulatory standards and were overseen by the institutional review board. Maximal tumor burden was not exceeded according to the institutional review board guidelines: for orthotopic tumors, mice were euthanized upon symptoms of distress, for subcutaneous transplantations, maximal tumor burden was 2cm^3. No sex bias was introduced during the generation of experimental cohorts.

Note that full information on the approval of the study protocol must also be provided in the manuscript.
